# Elephant‐Skin‐Inspired Porous Cementitious Tiles with Programmable Crack Networks for Passive Cooling

**DOI:** 10.1002/adma.202523133

**Published:** 2026-04-20

**Authors:** Qingya Huang, Kun‐Hao Yu, Ji Yoon Bae, Yunchan Lee, Dorit Aviv, Shu Yang

**Affiliations:** ^1^ Department of Materials Science and Engineering University of Pennsylvania Philadelphia Pennsylvania USA; ^2^ Department of Architecture Weitzman School of Design University of Pennsylvania Philadelphia Pennsylvania USA

**Keywords:** cementitious composites, elephant skin‐inspired, evaporative cooling, programmable fracture, substrate‐guided cracking

## Abstract

Passive evaporation of water reduces a building's cooling energy demand. However, water is often wasted due to rebound, uneven spreading, and rapid drainage. Here, we present an elephant‐skin‐inspired crack network architecture in porous diatomaceous earth (DE)‐cement composites to capture, route, and store water with minimal runoff. DE's micro/nanoporosity enables ultrafast (sub‐50 ms) water imbibition, while crack networks act as capillary conduits that redistribute water across and up inclined surfaces. Substrate‐guided stress concentration converts drying‐induced stochastic fractures into deterministic crack lattices that route and retain water on inclined surfaces, enabling geometry‐tunable, water‐efficient evaporative cooling. Tiles of hexagonal lattices with intermediate crack density maximize lateral redistribution and delay drainage. Infrared imaging reveals edge‐dominated evaporation, sustaining prolonged cooling. In a mockup home model covered with DE‐cement tiles, under repeated water dosing and IR heating, the temperature beneath the DE‐cement tiles is maintained at ∼32°C vs ∼42°C and ∼52°C for cracked and non‐cracked commercial stucco, respectively. The study offers a simple, scalable route for passive cooling.

## Graphical abstract

Elephant‐skin‐inspired crack networks are programmed in porous diatomaceous earth (DE)‐cement composites using substrate‐guided, stress‐concentration induced fracture. The resulting crack lattices act as capillary conduits that redistribute water, while the porous matrix stores moisture. This architecture enhances water retention and prolongs evaporative cooling, offering a scalable strategy to fabricate porous cementitious tiles for improved building‐envelope cooling performance.

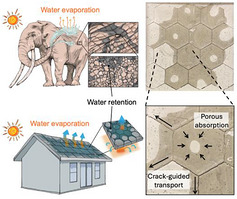

## 1 Introduction

90% of people spend about an average of 22 h indoors every day [1]. The building sector accounts for nearly 40% of the primary energy use in the United States, with about half of that energy dedicated to heating, cooling, ventilation (HVAC), and lighting [2]. Building envelopes play a pivotal role in reducing building energy consumption by attenuating the transfer of air, humidity, heat, and light between the exterior climate and the indoor space, while reducing the urban heat island effect. Various building envelopes have been proposed for passive cooling [3, 4, 5]. However, an energy expenditure for actuation and maintenance is necessary, and adaptive building envelopes remain largely in lab settings and are cost‐prohibitive. Approaches such as embedding water tubing and phase change materials in the wall [6], coating of evaporative cooling hydrogels [7, 8, 9], and radiative cooling materials face corrosion, high cost, and lack of outdoor durability. As rising temperatures further increase cooling demand, there is a clear need for envelope materials that deliver passive, durable, and scalable cooling.

By contrast, evaporative cooling is a passive strategy that exploits the latent heat of vaporization of water, requiring minimal external energy and offering low‐cost operation [10, 11, 12, 13]. This approach is particularly effective in dry and semi‐arid climates. In such environments, rainfall or intermittent water inputs can potentially be captured by the building envelope rather than immediately running off or evaporating, enabling passive cooling of façades without additional water consumption. While evaporative cooling has been widely studied at the material and laboratory scale, several projects have attempted to implement this strategy in the built environment. For example, the BioSkin façade system implemented in Sony City Osaki in Tokyo uses porous ceramic pipes with circulating rainwater to induce evaporative cooling on the building façade, contributing to localized microclimate cooling [14]. Similarly, the CoolAnt “Beehive” terracotta cooling façade employs porous clay modules with water circulation to passively cool the surrounding air through evaporation [15]. However, practical deployment faces persistent challenges. Hard water can introduce mineral fouling that clogs distribution lines and degrades pad performance, particularly in unconditioned or recycled‐water systems [16]. More fundamentally, many building‐grade exterior surfaces are water‐inefficient: droplets bounce or run off, spreading is uneven, and the wetted area transitions early from Stage I (capillary‐sustained) to Stage II (diffusion‐limited) evaporation [16, 17, 18]. Conventional cementitious and stucco surfaces, though inexpensive and ubiquitous, typically offer limited control over liquid pathways; stochastic cracking further compromises performance and durability. Current strategies oversupply water, which could lead to molding, or rely on coatings that cannot meet the typical building durability requirement for an outdoor use condition [19, 20]. While evaporative cooling is a long‐established strategy, scalable manufacturing of cementitious materials capable of efficiently capturing, retaining, and redistributing limited water inputs with designed transport pathways remains underdeveloped.

**FIGURE 1:**
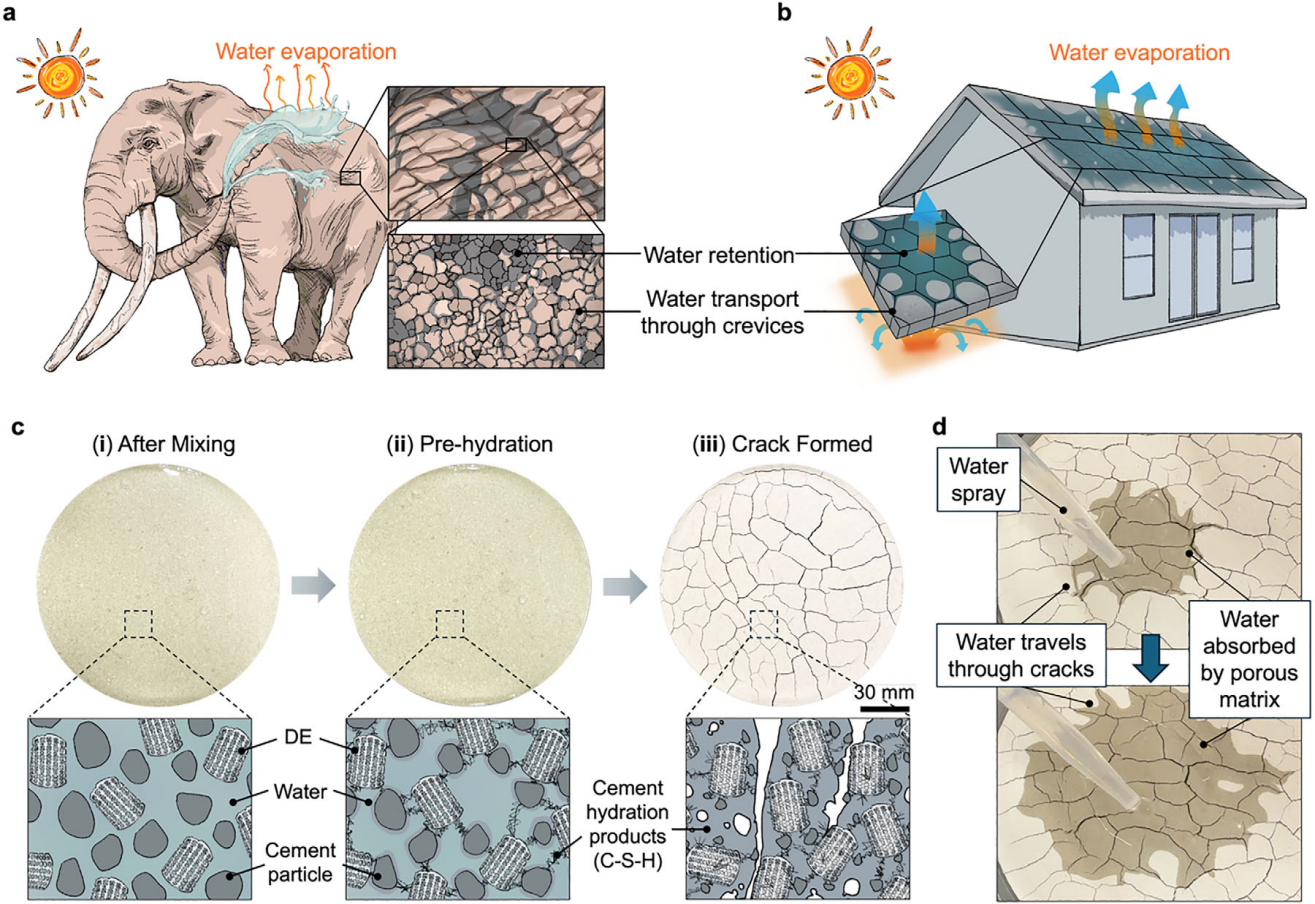
Bio‐inspired porous‐cracked tiles for water capture and transport. a) Schematic of an elephant spraying water to regulate temperature on tilted terrain; inset: wrinkled, creviced skin that retains water and guides flow. b) Concept of an elephant‐skin‐inspired porous tile with self‐formed crevices for water retention and directional transport. c) Photographs with corresponding schematics of crack formation: (i) freshly mixed DE‐cement paste cast as thin discs, (ii) 1 h pre‐hydration to initiate early bonding, (iii) heat‐induced drying and spontaneous cracking. d) Sequential images showing rapid water uptake and crack‐guided transport on the cracked DE‐cement sample.

Nature offers instructive alternatives. For example, elephant skin exhibits dense crack networks that could enhance water retention and redistribution, thereby promoting prolonged surface wetting relevant to evaporative heat loss [21, 22]. Rather than arising from stochastic shrinkage, these fractures result from stress localization imposed by the papillary dermis, which concentrates bending stresses as the stratum corneum thickens [21]. The resulting network facilitates capillary spreading and retains up to an order of magnitude more water than smooth skin, prolonging surface wetting and evaporative heat dissipation [21, 22, 23] (Figure 1a). Stress‐directed crack patterns also occur in soils, colloids, and concrete where drying shrinkage exceeds cohesive strength [24, 25, 26, 27, 28]. Prior studies show that guided crack initiation can yield microscale patterns without complex fabrication, enabling defect control in coatings and functional surface structuring [29, 30]. Recent studies have leveraged crack‐ or channel‐assisted capillary transport in porous metal systems for device‐level cooling applications such as flexible electronics [31]. While these systems exploit mesocracks that emerge from the material's microstructure during fabrication to enhance wicking, they do not deliberately program crack networks, nor do they do so in cementitious materials to control water capture, redistribution, and retention across large‐area surfaces. At the building scale, the system must (i) capture droplets within tens of milliseconds to suppress rebound; (ii) maintain a percolating capillary reservoir through programmed channels that direct lateral redistribution over edge drainage; and (iii) achieve these functions through a durable, low‐complexity, m^2^‐scale process compatible with outdoor wet‐dry and thermal cycling. Existing solutions trade off speed, retention, durability, and manufacturability.

Here, we introduce an elephant skin‐inspired, substrate‐guided cracking strategy in low‐cost, stucco‐like cementitious composite formulated with diatomaceous earth (DE) (Figure 1b). DE's micro/nanoporosity enables sub‐50 ms imbibition and rebound suppression, while the cement matrix provides mechanical robustness and compatibility with existing façades and roof renders. A brief pre‐hydration window forms hydration bridges that lock in a porous, cracked morphology upon re‐wetting. We quantify how capillary shrinkage and substrate friction set crack spacing/area, then emboss ridge patterns that act as stress concentrators, converting stochastic fracture to deterministic radial, parallel, or concentric networks. Scaling to tiles of 2D periodic lattices (triangular, square, hexagonal), an analysis of directional flow probabilities combined with IR thermography shows that an intermediate‐density hexagonal network maximizes lateral redistribution, delays drainage, and sustains edge‐dominated Stage I cooling at fixed water input. Finally, in a benchtop roof model that compares DE‐cement tiles with commercial stucco tiles (cracked and non‐cracked) under identical watering, the temperature beneath DE‐cement tiles remains ∼32°C vs ∼42°C (cracked stucco) and ∼52°C (non‐cracked stucco), respectively, directly linking the materials design to improved building‐envelope cooling performance.

## 2 Results

### 2.1 Capillary‐Driven Fracture Mechanism in DE‐Cement Composites

DE is a siliceous material composed of fossilized diatom frustules with perforated, often cylindrical shells [32]. Its hierarchical porosity yields a high surface‐area‐to‐volume ratio (Figure S1), low bulk density, and structural robustness, enabling rapid liquid uptake and strong moisture retention [33, 34]. The amorphous silica surface is inherently hydrophilic due to abundant silanol groups, which attract water molecules, while capillary forces within the micro‐ and nanoscale pores facilitate internal water storage. Under dry or heated conditions, the high surface area promotes efficient water evaporation through continuous wicking. Owing to these properties, DE is widely used in applications such as moisture regulation, filtration, and carbon capture [35, 36, 37]. We cast 1:1 (mass) DE‐cement paste into 3 mm‐thick, 150 mm‐diameter discs (Figure 1c(i)), allow 1 h pre‐hydration (Figure 1c(ii)), then dry at 60°C to induce spontaneous cracking (Figure 1c(iii)). The dried material exhibits a density of 740 kg/m^3^, substantially lower than that of a pure cement paste (∼1300 kg/m^3^) prepared using the same protocol. Adsorption‐desorption isotherms indicate pores down to ∼2 nm (Figures S1d and S2), attributed to DE. Upon spraying water onto the cracked sample, cracks act as conduits and the porous matrix rapidly imbibes water (Figure 1d; Movie S1). After full re‐saturation and immersion, the composite does not fall apart but retains its crack morphology and structural integrity.

**FIGURE 2:**
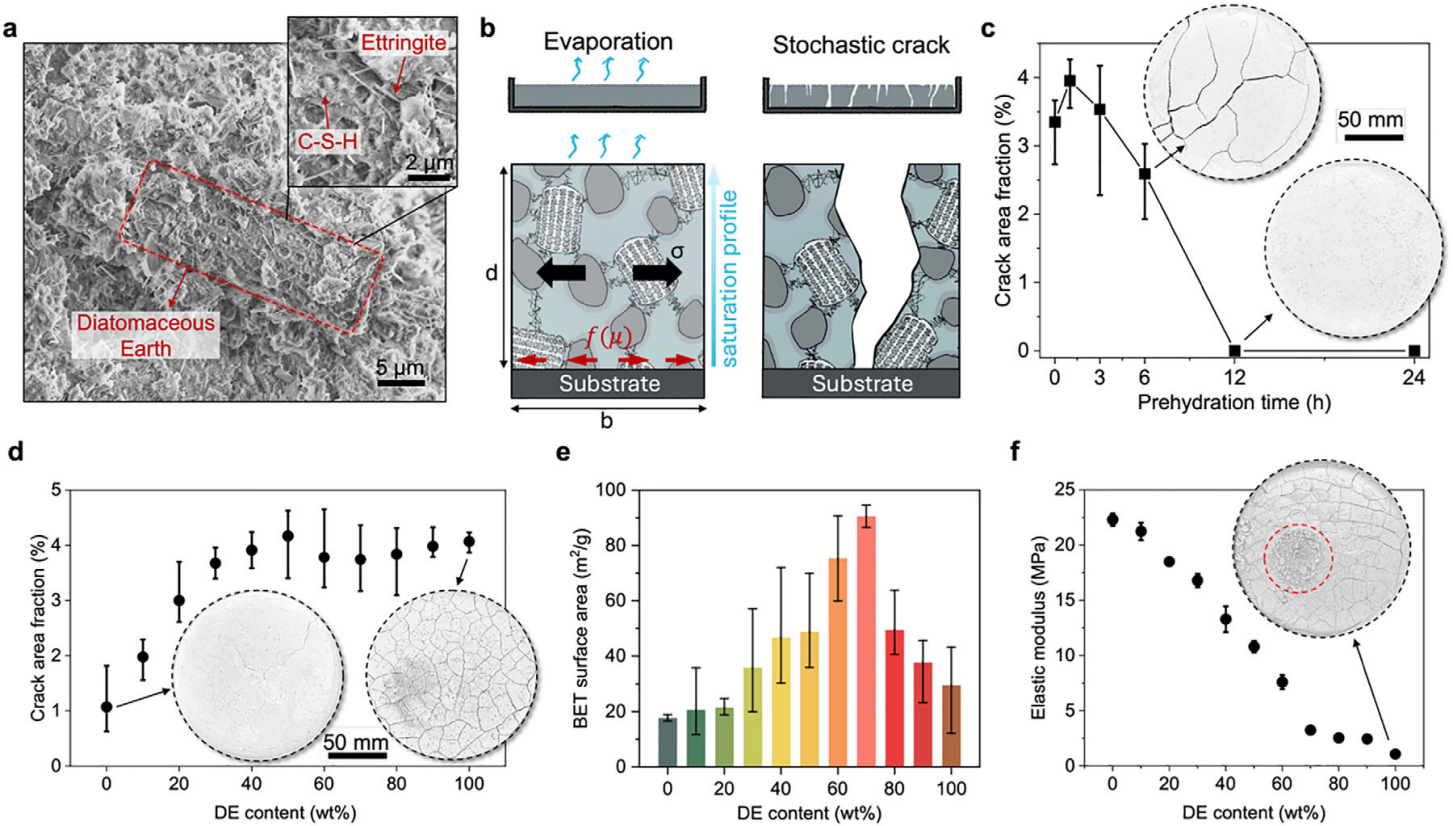
Early hydration, shrinkage‐driven cracking, and material property evolution. a) SEM images showing ettringite and C─S─H bonded to DE frustules (outlined in a red dashed rectangle). b) Schematic of drying‐induced shrinkage opposed by substrate friction, leading to tensile stress buildup and fracture. c) Crack area fraction vs pre‐hydration time. Inset: representative samples at 6h and 12 h, respectively. d) Crack area fraction vs DE mass ratio. Inset: representative samples at 0 and 100 wt.% DE. e) BET surface area vs DE mass ratio. f), Elastic modulus of dried, cracked samples vs DE mass ratio. Inset: disintegration of the 100 wt.% DE sample upon gentle water pipetting at ∼5 cm height.

The binder is ordinary Portland cement (OPC), whose principal clinker phases are tricalcium silicate (3CaO·SiO_2_, C_3_S), dicalcium silicate (2CaO·SiO_2_, C_2_S), tricalcium aluminate (3CaO·Al_2_O_3_, C_3_A), and tetracalcium aluminoferrite (4CaO·Al_2_O_3_·Fe_2_O_3_, C_4_AF). Commercial OPC is introduced with a small amount of calcium sulfate (typically gypsum) to moderate the very rapid C_3_A reaction and prevent flash set. During the initial 1 h prehydration period, C_3_A in the cement reacts with calcium sulfate to form ettringite, a needle‐like calcium sulfoaluminate hydrate (3CaO·Al_2_O_3_·3Ca(SO_4_)·32H_2_O). Concurrently, the hydration of C_3_S begins, producing incipient calcium silicate hydrate (3CaO·SiO_2_·3H_2_O, C‐S‐H) gel and calcium hydroxide (Ca(OH)_2_) [38]. The internal water reservoirs within the porous DE establish localized ionic concentration gradients (e.g., Ca^2+^, SiO_4_^4−^) that drive heterogeneous nucleation of hydration products at the DE‐cement interface, promoting a physically interconnected microstructure [37]. Upon drying, evaporation induces capillary menisci that generate tensile stresses within the solid skeleton, leading to local shrinkage [39, 40]. Meanwhile, early hydration produces C─S─H and ettringite that bridge the DE‐cement interface (Figure 2a), imparting sufficient cohesion to resist redissolution upon re‐wetting (Figure 1d).

Desiccation cracking of the thin DE‐cement discs (1–3mm thick) is primarily governed by two competing forces: tensile contraction during drying and interfacial friction between the substrate and the shrinking disc [26, 41]. As surface water evaporates, negative capillary pressure induces internal contraction of the prehydrated DE‐cement; however, physical confinement of the substrate leads to friction at the bottom interface, which resists shrinkage and results in stress accumulation across the disc. When this stress exceeds the tensile strength of the composite, cracking occurs. This mechanism is validated by a control experiment by spray coating a non‐stick lubricant onto the substrate, followed by casting DE‐cement on top: in the absence of interfacial friction, the sample shrinks freely into a smaller disc without cracking (Figure S3) [42]. We model the system as a 2D disc constrained by the substrate (Figure 2b). The frictional resistance acting along a crack segment of length *b* can be expressed as:

(1) $$F_{friction}=f\left(\mu \right)\cdot b$$

where *μ* is the friction coefficient, *b* is also referred as the crack spacing, and *f*(*μ*) is the frictional force per unit length. As the material undergoes drying‐induced contraction, the restrained bottom surface generates a tensile stress gradient along the thickness *d*. Assuming uniform stress distribution for a thin disc, the average tensile stress across *d* is:

(2) $$\sigma =\frac{F_{friction}}{d}\;$$

Cracks occur when the stress exceeds the material's fracture strength σ_*c*_,

(3) $$\sigma \geq \sigma_{c}$$

Substituting Equation (1) into Equation (2) gives the critical condition for crack spacing:

(4) $$\;\sigma_{c}=\frac{f\left(\mu \right)\times b}{d}\;$$

This relation implies that, for fixed friction and fracture strength, *b* and *d* have a linear relationship. Experimental observations confirm this trend: as *d* increases, *b* widens, resulting in larger fragments and a lower crack density (Figure S4). Crack formation is governed by the balance between capillary shrinkage stress σ, induced during evaporation, and the fracture strength σ_*c*_, determined by interparticle bonding. When σ exceeds σ_*c*_, cracking occurs. In our system, extending the pre‐hydration period enhances interparticle cohesion via continued cement hydration, thereby increasing σ_*c*_. Consistent with this mechanism, we observe a progressive reduction in crack area fraction of the sample as the pre‐hydration time is prolonged, with crack‐free samples achieved after 12 h pre‐hydration, indicating extensive bonding (Figure 2c; Figures S5–S7). Crack area fraction is the total crack area divided by the sample's surface area.

### 2.2 Influence of DE Mass Loading on Crack Evolution and Material Properties

Beyond prehydration time, DE loading influences both σ and σ_*c*_. We systematically vary the DE mass fraction from 0 (pure cement) to 100 wt.% (pure DE). The crack area fraction initially increases with increasing DE wt.%, but plateaus beyond 50 wt.% DE (Figure 2d; Figure S8), indicating a shift in the dominant cracking mechanism from cement hydration‐controlled to one governed by particle packing. At low DE, rapid early hydration generates a cohesive matrix that resists shrinkage‐induced cracking, resulting in a low crack fraction (∼1%). However, as DE is introduced, early hydration is delayed, leading to increased porosity and reduced initial binding strength, which enhances susceptibility to desiccation‐induced shrinkage stresses. Moreover, the intrinsic porosity of DE promotes capillary‐driven water migration and accelerates evaporation, amplifying internal stress and facilitating crack formation. At DE mass fractions ≤50 wt.%, the crack area fraction continues to increase; as the cement content decreases, the pre‐hydration products that contribute to matrix cohesion are also progressively reduced. However, at >50 wt.% DE, interparticle cohesion (silanol‐mediated hydrogen bonding/capillary bridges) and packing friction inhibit the formation of new cracks, leading to a plateau in crack density.

Dry density measurements reveal a progressive reduction with increasing DE wt.%, as the composite becomes lighter and more porous (Figure S9). However, Brunauer‐Emmett‐Teller (BET) surface area measurements display a non‐monotonic trend: the surface area peaks at 70 wt.% DE (Figure 2e), which can be attributed to two competing effects: (i) the inherent porosity of DE, which increases the total pore volume with rising DE loading, and (ii) cement hydration products, which could contribute additional nano‐ and mesopores that enhance surface area. These two effects synergistically increase capillary porosity with DE loading up to 70 wt.%. Beyond this point, the cement phase becomes insufficient to bridge neighboring DE particles, resulting in the formation of isolated voids rather than a continuous porous network (Figure S10).

The mechanical properties of the DE‐cement composites are critical because they determine the integrity of the porous network during handling, transport, and repeated wetting‐drying cycles. In particular, the strength and stiffness arising from interparticle bonding govern whether the material can absorb and retain water without structural collapse. To evaluate mechanical properties, we perform micro‐indentation analysis on the dried, cracked samples with varying DE wt.%. At and beyond 70 wt.% DE, the elastic modulus of the composite (*E*) drops below 5 MPa, indicating that the interparticle interactions are predominantly governed by weak van der Waals forces and residual hydrogen bonding rather than cementitious bonding (Figure 2f). At 100 wt.% DE, the sample disintegrates upon re‐wetting, confirming the absence of a binding phase. In contrast, at 50 wt.% DE, the composite retains its original crack morphology after re‐wetting over 10 cycles, demonstrating that residual hydration products maintain sufficient structural integrity (*E* > 10 MPa) to preserve the network architecture while enabling rapid water absorption (Figure 1d). Consistent with this observation, the 50 wt.% DE composite exhibits an average compressive strength of approximately 2.9 MPa at 3 days (Figure S11), which would increase further as curing continues. Importantly, this early‐stage value is already comparable to those of cement‐based plaster and render materials used in building envelopes (3–10 MPa) [43, 44], indicating sufficient mechanical integrity for the intended non‐load‐bearing façade or tile applications.

### 2.3 Capillary and Crack‐Enabled Water Retention and Transport

**FIGURE 3:**
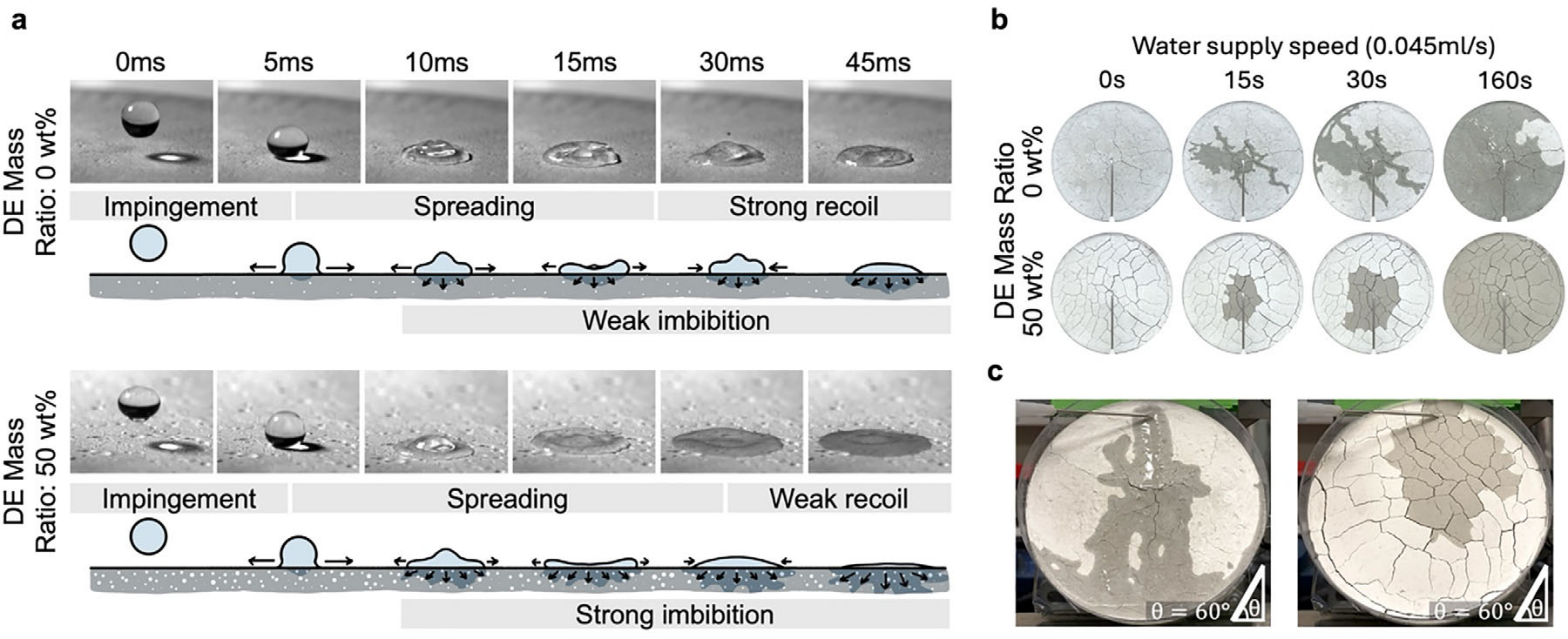
Droplet impact, continuous flow, and tilt‐retention enabled by porosity and cracks. a) High‐speed images of a water droplet impacting material surfaces with 0 and 50 wt.% DE, with schematics of impingement, spreading, recoil, and imbibition processes. b) Time‐lapse images of constant water delivery at the center of the cracked samples (0 and 50 wt.% DE) showing lateral distribution vs surface pooling. c) Photographs of the cracked samples (0 and 50 wt.% DE) inclined at 60° under continuous water input at the top edge, highlighting uphill water wicking and retention in the DE‐rich sample.

Sustained evaporative cooling requires suppressing water rebound/runoff and providing residence time for imbibition. To probe early‐stage wetting and transport behaviors, we perform high‐speed imaging to capture droplet impingement on the dry samples with 0 and 50 wt.% DE, respectively. The 0 wt.% DE sample undergoes a classic impingement‐spreading‐recoil‐rebound sequence, with minimal absorption and pronounced upward jetting observed at 30 ms. In contrast, the 50 wt.% DE sample displays significantly enhanced lateral spreading (1.6× in width), followed by rapid absorption within 45 ms, three times faster than that of the 0% DE sample (Figure 3a; Movie S2). This behavior arises from capillary imbibition into the DE‐rich micro/nanoporous network, where the combined effects of hierarchical porosity and the intrinsically hydrophilic silanol surface dissipate the droplet's kinetic energy and suppress rebound through inertial‐capillary arrest [17]. Because droplet impact conditions (height, size, and fluid properties) are held constant, the observed differences in spreading and absorption arise solely from substrate properties, namely porosity, surface energy, and internal capillarity. These absorption dynamics are consistent with the Washburn Equation (5) [45]:

(5) $$t_{I}=\frac{2\eta L^{2}}{\gamma r_{P}\cos\theta }\;$$

where *t_I_* is the imbibition time, *η* is the liquid viscosity, *L* is the penetration depth, *γ* is surface tension, *r_P_* is the effective pore radius, and *θ* is the apparent contact angle. With fixed *η* and *γ*, the DE‐rich matrix promotes faster absorption due to its larger *r_P_* and lower *θ*. As a result, the liquid front reaches the same penetration depth in a much shorter time, yielding the observed sub‐50 ms penetration time. This rapid imbibition supports moisture retention and enhanced evaporation, making the material well‐suited for evaporative cooling.

Building on droplet‐scale wetting behavior, we next investigate continuous water transport and retention under steady‐state input conditions. A controlled water flow is introduced at the center of the dry samples using a syringe pump, mimicking persistent water exposure (e.g., rainfall). Optical imaging reveals that in 50 wt.% DE‐cement, water is rapidly absorbed at the point of contact and transported radially outward through the crack network, enabling sustained uptake and uniform wetting across the sample. In contrast, the pristine cement sample exhibits limited absorption and surface pooling (Figure 3b; Movie S3). To visualize subsurface water movement, we heat the sample to 60°C, then introduce room‐temperature water, and use infrared (IR) imaging to track the flow (Figure S12). The resulting temperature gradient enables clear visualization of water propagation, which initially flows through the cracks as the primary conduits and is subsequently absorbed into the surrounding porous matrix (Figure S13a; Movie S4). In contrast, the pristine cement sample shows markedly slower absorption kinetics; despite cracks aiding flow, the adjacent matrix does not effectively uptake water, causing liquid to bypass the bulk material and accumulate at the sample edges (Figure 3b, S13a, Movie S4). A direct comparison between optical and IR images at the same time point (60 s) further highlights this behavior (Figure S13b): the pristine cement sample exhibits extensive surface pooling with limited subsurface transport, whereas the DE‐cement composite shows minimal surface accumulation as water is rapidly redistributed through the crack network and absorbed into the porous matrix. These observations demonstrate a synergistic interaction between matrix porosity and crack‐assisted capillary transport. The high surface area and pore connectivity of the DE‐rich matrix facilitate rapid lateral imbibition, while the cracks act as the vasculature networks that distribute water efficiently across the entire structure. This combined mechanism enables persistent water uptake and retention, even when the substrate is tilted. When inclined at a 60° angle, the 50 wt.% DE sample effectively retains water without runoff, demonstrating uphill wicking and surface adhesion, which mimics the capillary anchoring and surface patterning strategies observed in elephant skin. In contrast, the 0 wt.% DE sample exhibits rapid flow along the tilted substrate with poor retention (Figure 3c; Movie S5).

### 2.4 Bio‐inspired Substrate Patterning for Programmable Crack Control

**FIGURE 4:**
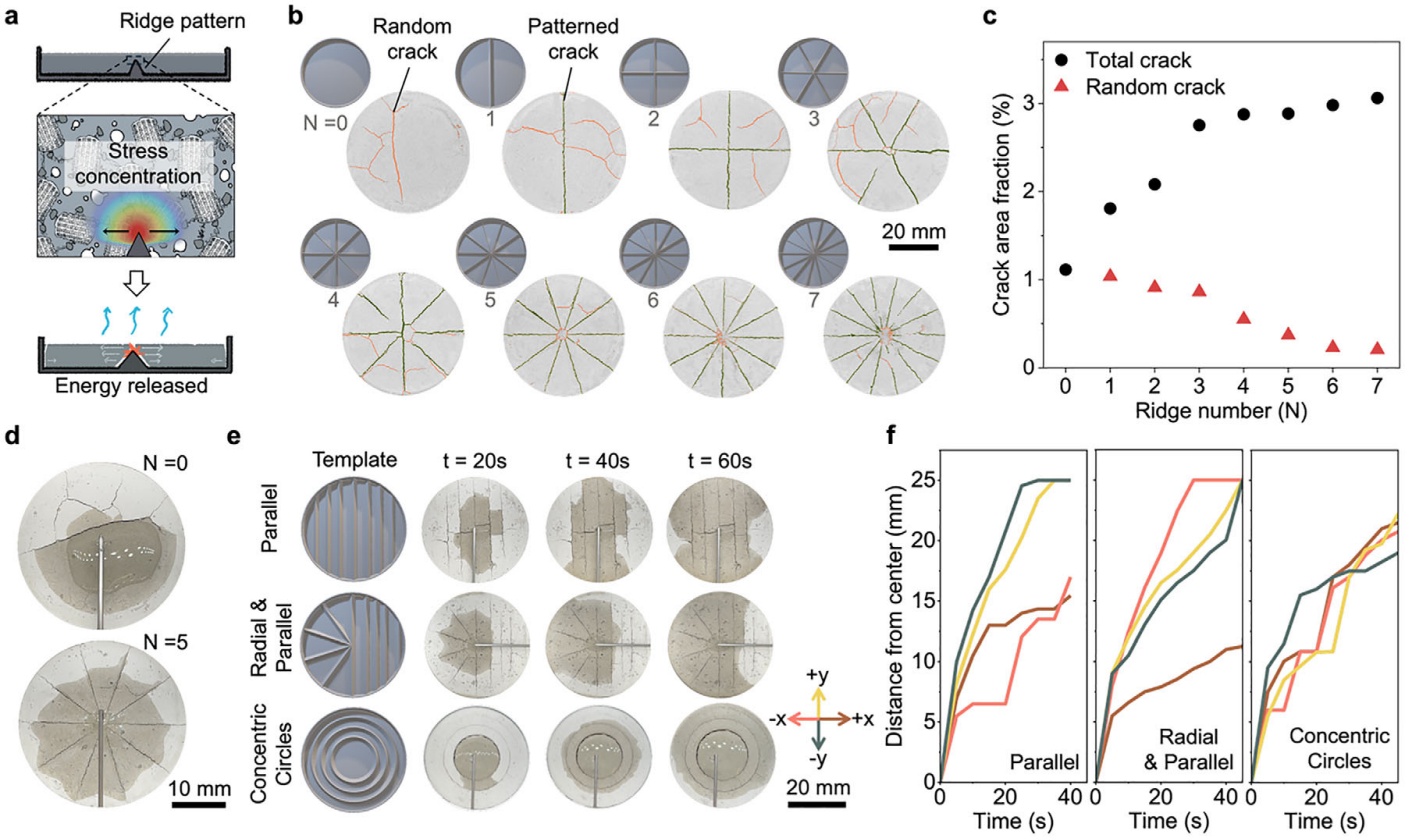
Substrate‐guided crack programming and directional transport. a) Schematic of the triangular ridge features on the substrate concentrating tensile stress and initiating guided cracks. b) Top‐view images of discs formed on substrates with ridge numbers *N* = 0–7 and the corresponding CAD models. Cracks not aligned with the designed paths are classified as random (highlighted in red). c) Total and random crack area fractions vs the ridge number. d) Continuous water input on *N* = 0 and *N* = 5 samples, showing enhanced radial distribution with guided cracks. e) CAD models for three substrate geometries – parallel ridges, radial & parallel hybrid, and concentric circles – and time‐sequence images showing water spreading under a constant water delivery at the center of the disc. f) Water propagation distance vs time along +x, –x, +y, and –y directions for each geometry.

In natural systems, crack initiation is often coupled to mechanical cues encoded within the underlying structure. Elephant skin provides an illustrative example: its crack networks have been shown to arise from stress localization imposed by dermal papillae beneath a thickening keratinized layer, rather than from frustrated shrinkage [21]. Valleys between papillae function as mechanical stress concentrators, channeling bending stresses that reproducibly guide crack propagation. Translating this strategy, we implement a substrate‐guided cracking approach to program fracture pathways when drying the composites. Triangular ridge patterns embossed on the mold base serve as geometric stress concentrators, amplifying tensile stresses at ridge tips (Figure 4a). This drives crack initiation along the predefined paths, leading to deterministic crack lattices.

To evaluate the efficacy of this approach, we 3D print circular molds with varying numbers of radial ridges (*N*), from 0 (flat control) to 7 (each segment area = $\frac{\pi r^{2}}{14}$). In the absence of ridges, cracks form randomly across the surface, as indicated by red‐highlighted fracture lines (Figure 4b). As *N* increases, cracks increasingly align with the ridge pattern, initiating directly above the ridge tips (Figure 4a). At a low *N* (e.g., 1–2), the patterned cracks emerge but do not fully relieve the drying‐induced shrinkage stress, resulting in secondary random cracks. When *N* ≥ 5, the shrinkage energy is primarily released along the programmed paths, and the area fraction of non‐programmed (random) cracks drops below 0.2% (Figure 4c). To quantify spatial fidelity, we compare the areas of the crack segments to their corresponding predefined segment areas imposed by the ridge geometry. Increasing *N* yields a tighter clustering of the measured crack segment areas around the predefined segment area value, confirming enhanced uniformity and control in crack segmentation (Figure S14). Notably, the total crack area (guided + random regions) increases with *N* and plateaus at ∼3% when *N* > 5, vs ∼1% when *N* = 0. This suggests that without the guided fracture mechanism, substantial drying‐induced shrinkage stress remains stored in the material, which could lead to material failure when additional stress is introduced, such as during cycles of wetting and drying. To confirm the presence of residual stress in a non‐patterned sample (*N* = 0), we subject the sample to 50 repeated wetting‐drying cycles. Additional cracks emerged during cycling (Figure S15a), indicating that drying‐induced shrinkage stresses remained stored in the material and are subsequently released through delayed fracture. In contrast, ridge‐patterned samples exhibit deterministic crack lattices that remain unchanged after 50 cycles (Figure S15b), demonstrating that the programmed crack network effectively accommodates the shrinkage strain and stabilizes the fracture pattern. We further evaluated the stability of the programmed crack networks under prolonged water exposure by immersing the patterned samples in water for 24 h, followed by drying. Optical inspection confirmed that the crack geometry and composite matrix remained unchanged after immersion (Figure S15c), supporting the structural stability of the crack‐programmed architecture under extended saturation.

### 2.5 Mechanism of Substrate‐Guided Crack Programming

When drying DE‐cement composites, random cracks could arise from non‐preferential water evaporation from the surface layer in competition with substrate‐induced stress restraint Equations ((1), (2), (3), (4)), especially when the film is thick (Figure S4). This is because evaporation proceeds primarily from the top surface, generating a vertical saturation gradient (Figure 2b). This drives drying‐induced shrinkage in the upper layers, which is resisted by the underlying substrate, producing a tensile stress gradient across the film thickness. In a non‐patterned system, stress accumulates isotropically, and fractures nucleate stochastically. However, when ridge patterns are incorporated into the substrate, the geometry locally amplifies stress near the ridges, enabling programmed crack formation [46], which is initiated at the preselected locations (Figure 4a). As evaporation progresses and the internal stress reaches a critical threshold, ridge tips act as stress concentrators. Cracks initiate at these ridge tips and propagate upward, releasing stored strain energy. As drying continues, the cracks extend downward toward the substrate, completing the through‐thickness fracture. This mechanism follows classical fracture mechanics via the stress concentration factor (SCF), *K_t_*, which quantifies the amplification of the local stress at geometric discontinuities. The local stress at a ridge tip is given by:

(6) $$\sigma_{tip}=K_{t}\;\cdot \sigma$$

For a triangular notch, *K_t_* increases as the ridge tip angle *α* decreases, approximated by:

(7) $$K_{t}\approx 3+\frac{2}{\sin\left(\frac{\alpha }{2}\right)}$$

This equation shows that as the *α* decreases (i.e., the notch becomes sharper), *K_t_* increases, leading to stronger localization of stress at the ridge tip. In Figure 4b, we systematically increased *N* on the substrate from 1 to 7. To ensure that the total ridge volume remained constant across all samples, we vary the triangular ridge tip angle between 15° and 45°, such that adding more ridges does not increase the total volume beneath the sample. This design maintained a consistent material height above the ridge tips, thereby keeping the applied drying‐induced stress *σ* at the ridge location comparable across all conditions (Equation 6). To investigate the effect of *α* on crack patterning, we conduct a control experiment using two molds with the same *N* = 6, but different ridge tip angles (15° and 45°) (Figure S16). Despite the difference in geometry, both samples produce similar crack area fractions with well‐aligned, guided cracks, indicating that variations in *α* within this range did not substantially affect pattern fidelity. This outcome arises because *K_t_*, which scales sharply with *α*, remains sufficiently high in both cases to exceed the material's fracture strength σ_*c*_. Once surpassing the critical stress threshold for crack initiation, small differences in *K_t_* become less influential. In contrast, substrates with rectangular and concave triangular (V‐shaped) grooves fail to initiate guided cracks (Figure S17), emphasizing the necessity of sharp geometric features for effective crack localization. Because material volume and drying conditions are fixed, the total drying‐induced strain energy is comparable across various patterned samples. As *N* increases (i.e., as more cracks are pre‐programmed into the system), this strain energy is distributed over a larger number of fracture sites. Consequently, the energy released at each crack is reduced, leading to narrower crack widths (Figure S18).

This strategy enables the deterministically controlled formation of cracks, allowing for programmable water transport and absorption. To demonstrate the effect of crack geometry on fluid transport, we continuously drip water at the center of the *N* = 0 and 5 samples, respectively (Figure 4d, Movie S6). When *N* = 0, water initially infiltrates the porous matrix near the impact site, but the absence of oriented cracks leads to inefficient lateral transport. As the pores in the central region saturate, excess water accumulates on the surface, limiting further distribution. In contrast, *N* = 5 sample with radial cracks is able to rapidly direct water outward from the center, leveraging crack channels as the macro‐scale conduits. This facilitates nearly uniform spreading and sustained absorption across the entire structure, demonstrating that crack geometry can be engineered to enhance transport efficiency under localized water input, which is particularly advantageous for applications such as passive evaporative cooling, where water must efficiently cover the surface. To further illustrate the versatility of programmable fracture networks, we design three additional substrate geometries to guide water along distinct pathways: (i) parallel ridges aligned along the *y*‐axis, (ii) a hybrid geometry with parallel ridges on one half and radial ridges on the other, and (iii) concentric circular ridges (Figure 4e). In the sample with parallel patterns, water preferentially propagates along the pre‐aligned cracks in the *y*‐direction, while the lateral spreading in the orthogonal x‐direction is suppressed due to the absence of transverse cracks (Figure 4f; Movie S7). In the hybrid geometry sample, water moves rapidly outward along the radial side (−x, +y, −y directions), but is restricted on the parallel side (+x direction), revealing spatial asymmetry in transport behavior. The concentric circular pattern confines water within each ring, delaying outward progression until saturation is achieved in the inner zones. This produces a stepwise wetting profile along four orthogonal directions. These demonstrations show that substrate geometry not only controls the initiation and orientation of cracks but can also be engineered and programmed to influence the rate and path of fluid movement.

### 2.6 Scalable Periodic Crack Networks for Enhanced Water Retention and Distribution

**FIGURE 5:**
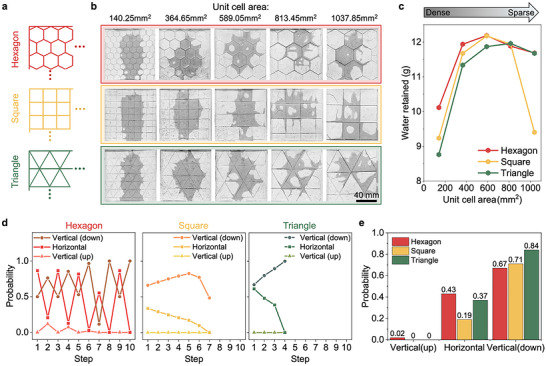
Periodic crack networks for scalable water retention and redistribution. a) Illustrations of the tessellated crack networks: triangular, square, and hexagonal. b) Screenshots at t = 10 s showing wetted areas on 60°‐tilted tiles for five unit cell areas per geometry under the constant water delivery at the top‐center of the sample. c) Water retained on 60°‐tilted tiles as shown in (b). d) Random‐walk simulations initialized at the top‐center using the directional flow probabilities measured at intersections (from Figure S21) for the intermediate density of each geometry. e) Averaged directional flow probabilities at pattern intersections.

While substrate‐guided cracks enable deterministic control at the disc scale, translating the concept to building envelopes – the roofs and façades that exchange heat and moisture with ambient air – requires patterns that are scalable, repeatable, and compatible with low‐complexity fabrication. Here, we design periodic lattice networks using three tessellatable unit cell geometries – triangular, square, and hexagonal – that extend seamlessly over large areas without geometric discontinuities (Figure 5a). We implemented these geometries as ridge patterns on 3D‐printed square substrates to cast square tiles with programmed lattice cracks; the square format simplifies stacking, alignment, and edge‐to‐edge joining without gaps. For each geometry, we cast five unit‐cell areas – 140.25, 364.65, 589.05, 813.45, and 1,037.85 mm^2^ – of varying densities (Figure S19). The smallest unit‐cell area matches the measured area of a single wedge‐shaped segment in the *N* = 7 radial specimen (segment area = $\frac{\pi r^{2}}{14}$ Figure 4b), a scale that effectively dissipates drying stresses. We then increase the unit‐cell areas stepwise to probe the onset of spontaneous, unprogrammed cracking within each geometry. (Figure S19). As the unit‐cell area increases, the programmed ridge boundaries become progressively less effective at redistributing shrinkage‐induced tensile stresses within each domain. Beyond a geometry‐dependent critical size, residual tensile stress accumulates in the interior of the unit cell, triggering spontaneous, unprogrammed cracks. This behavior establishes a practical upper bound on the unit‐cell size required to maintain pattern fidelity within each lattice geometry.

To assess the role of geometry and density, we drip a fixed volume of water (13.5 mL) onto the top of each inclined sample (60°) and quantify water retained on the sample after full delivery (Figure 5b,c; Figure S20, Movie S8). The bottom edge of the tile is left open to allow free drainage. Across all geometries, water uptake shows a non‐monotonic dependence on the unit cell size: intermediate patterns consistently yield the highest water retention, whereas both sparse and overly dense patterns reduce retention (Figure 5c). In the densest patterns, limited unit cell volume and narrow cracks lead to rapid local saturation and premature vertical drainage with minimal lateral spreading. Conversely, in sparse patterns, wide cells initially promote lateral flow, but long, straight cracks create unimpeded drainage paths, while absorption from crack channels into the bulk is restricted by slow diffusive transport. The sparse patterns outperform the denser ones, underscoring the importance of balancing crack connectivity with lateral redistribution. Water dosing from the upper edge of inclined samples, rather than from the center, similarly results in lateral redistribution through the programmed crack networks, indicating that the redistribution mechanism is not limited to a specific dosing location.

Among the three geometries, the hexagonal network consistently outperforms the triangular and square ones across all densities. To elucidate this behavior, we analyze water flow at the intermediate density, where uptake is maximal. Droplets are deposited at unit cell intersections, and the resulting flow probabilities in vertical and lateral directions are recorded (Figure S21a). These local probabilities are then used to simulate random‐walk paths starting at the top center (Figure S21b). On average, water requires ∼10 steps to exit the hexagonal network, compared to 7 for square and 4 for triangular patterns (Figure 5d; Figure S21b). The hexagonal geometry enables alternating vertical and lateral flow, producing zigzag propagation that delays drainage and maximizes surface coverage. By contrast, triangular and square lattices show strong downward bias with flow probability (*p*) of 0.84 and 0.71, respectively, while hexagons favor lateral transport (*p*, 0.43) with slight upward movement (*p*, 0.02) (Figure 5e). These results indicate that the hexagonal periodic network, at intermediate unit cell size, achieves the highest water retention among the tested designs by enabling multidirectional redistribution and prolonged residence time. During the repeated experimental uses, the same intermediate‐density tiles undergo more than 10 wetting‐drying cycles without additional cracking, indicating that the programmed lattice is stable under cyclic hydration‐drying processes.

### 2.7 Evaporative Cooling Performance of Periodic Crack Tile

**FIGURE 6:**
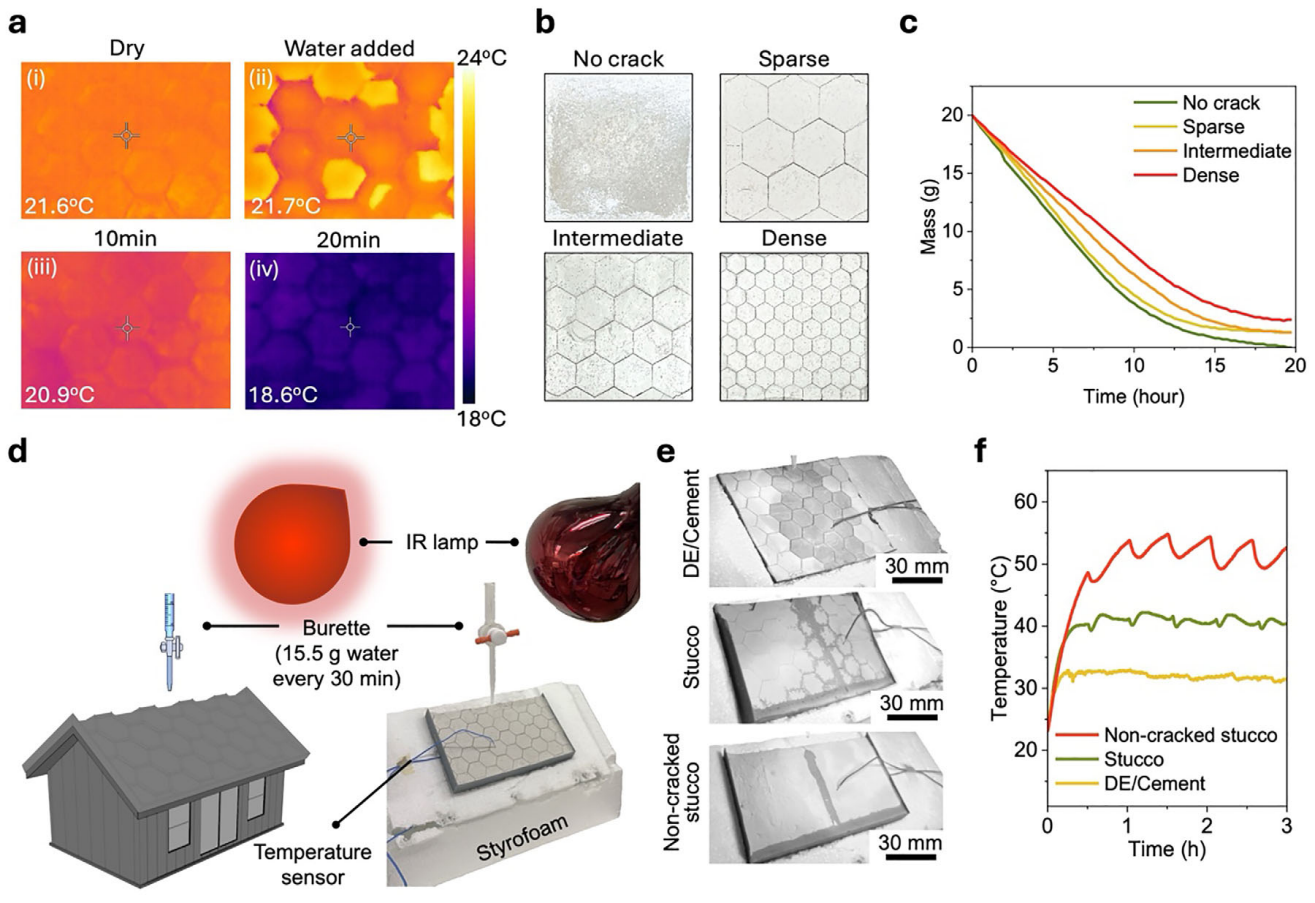
Evaporative cooling dynamics and model‐house demonstration. a) IR images of an intermediate‐density hexagonal tile at stages: (i) dry baseline, (ii) immediately after adding room‐temperature water, (iii) 10 min after adding room‐temperature water, (iv) 20 min after adding room‐temperature water. b) Images of the hexagonal tiles with varying unit cell sizes and a crack‐free control for the evaporation test. c) Mass loss vs time for the hexagonal tiles with varying unit cell sizes and a crack‐free control. d) Schematic and experimental setup for the model house demonstration. e) Water retention behavior on a DE‐cement tile with a hexagonal crack network, stucco tiles with and without cracks under identical water dosing. f) Temperature evolution measured beneath each tile over six watering cycles (3 h total).

We next evaluate the evaporative cooling performance of hexagonal periodic tiles using IR thermography, tracking temperatures during absorption, redistribution, and drying stages (Figure 6a). Before adding water, the tile exhibits a uniform baseline temperature of 21.6°C (Figure 6a(i)). Upon applying room‐temperature water, cracks first distribute liquid, while the porous DE‐cement matrix within each hexagonal cell imbibes water. This produces a transient thermal gradient across the tile, with cooler temperatures near the wetted perimeters (20.3°C) and a warmer center (21.6°C) that progressively equilibrate over time (Figure 6a(ii)). After 10 min, the sample equilibrates: water is uniformly distributed, the crack channels and matrix have comparable temperatures (edge: 20.9°C vs center: 21°C), and Stage I evaporation begins, characterized by constant, capillary‐sustained vaporization across the surface (Figure 6a(iii)). After 20 min, IR imaging reveals distinct, colder bands along the crack edges, with edge regions at 18.4°C compared to the interior at 19.0°C, indicating a localized enhancement of evaporation (Figure 6a(iv)). This behavior arises because the interior regions of each unit cell lose surface connectivity and transition to Stage II (diffusion‐limited) evaporation [47], while the perimeters remain supplied by capillary pathways connected to the cracks. At these three‐phase boundaries, reduced external resistance and persistent liquid films sustain high local flux, producing edge‐dominated evaporation and pronounced cooling along crack walls [48].

To examine how the edge effect scales with crack density, we compare three hexagonal tiles with different unit cell sizes, along with a crack‐free control of identical composition (Figure 6b). All samples are loaded with equal water volume and tested under natural convection at fixed temperature and relative humidity. Mass loss is continuously recorded, while both surface and subsurface temperatures are monitored. All samples exhibit similar subsurface cooling. However, evaporation rates diverge: the crack‐free tile loses water most rapidly, followed by the sparse pattern (large unit cells), whereas the dense hexagonal network evaporates slowest (Figure 6c). Despite higher local flux along edges, patterned tiles evaporate more slowly overall because only a fraction of the surface remains persistently wetted in Stage I. Increasing pattern density further suppresses bulk evaporation: although more edges remain active, the denser network sustains a cooler, more humid microclimate that lowers the effective vapor‐pressure gradient and slows overall water loss. With equal amounts of absorbed water and comparable total latent cooling energy, the differences lie in the cooling dynamics. The crack‐free sample evaporates water rapidly. In contrast, dense hexagonal networks moderate the instantaneous evaporation rate while sustaining a surface temperature below ambient air temperature for up to 20 h, thereby extending the effective cooling period with the same water input. These findings reveal that crack architecture not only governs water retention but also tunes the temporal profile of evaporative cooling. Evaporative cooling performance depends on environmental conditions such as ambient temperature and relative humidity, which determine the vapor‐pressure gradient driving evaporation. In this study, experiments were conducted under controlled laboratory conditions with fixed ambient temperature and humidity to enable direct comparison among different crack‐network geometries. While these factors influence the absolute magnitude of cooling, the relative performance trends reported here primarily reflect differences in water redistribution and retention governed by the crack architectures.

To demonstrate practical performance, we build a benchtop model home with tiles mounted on expanded polystyrene for potential use as building coatings, an IR lamp as the heat source (hot‐side surface, 42±1°C), and a constant water dose (15.5 mL per cycle) is applied at the ridge every 30 min. One thermocouple monitors the heat‐source surface; a second one is placed beneath the tile to record the transmitted temperature in real time (Figure 6d). As controls, we fabricate two sets of tiles with the same geometry as the DE‐cement composite from commercial stucco, using the same process: (i) hexagonal, cracked, and (ii) non‐cracked. As shown in Figure 6e, DE‐cement tiles spread and retain water within the porous‐cracked network, whereas the cracked stucco tile directs water through the cracks but drains quickly; the non‐cracked stucco tile spreads little and absorbs poorly. Commercial cement‐based stucco is selected as the reference material because it represents a widely used exterior façade finish in residential construction, thereby serving as an application‐relevant benchmark. Over six watering cycles, the temperature beneath the DE‐cement tile remains nearly constant at ∼32°C, whereas it rises to ∼42°C under the cracked stucco tile and ∼52°C under the non‐cracked stucco tile (Figure 6f). The DE‐cement tile maintains a stable temperature throughout repeated wet‐dry cycles, while both stucco samples exhibit strong oscillations (each watering produces a brief cooling followed by a rapid rebound as the thin surface water evaporates). Consequently, heat accumulates within the stucco tiles, leading to a persistent temperature increase of 10–20°C higher than that of the DE‐cement tile. These results show that, with the same water input, the bio‐inspired DE‐cement tile sustains prolonged evaporative cooling through effectively increasing water retention, demonstrating its promise as a scalable, water‐efficient material for passive cooling applications. A schematic illustration of representative roof‐scale water‐feeding configurations shown in Figure S22 suggests how the programmed crack networks could enable efficient surface wetting with larger spacing between supply points, thereby reducing water consumption compared to conventional cementitious surfaces.

## 3 Conclusion

We demonstrate that elephant skin‐inspired, substrate‐programmed crack lattices transform a drying weakness into a capillary asset for water‐efficient evaporative cooling. In porous cementitious composites made from DE‐cement, a short pre‐hydration window establishes hydration bridges that lock in the porous‐cracked morphology upon re‐wetting, while a friction‐mediated balance between the capillary shrinkage and fracture strength sets a predictive spacing‐thickness relation for the resulting crack networks. Embedding sharp geometric concentrators routinizes fracture, suppresses randomness, and channels liquid along designed paths. When scaled to tiles (100 mm × 100 mm) of periodic crack architectures, intermediate‐density hexagonal lattices maximize lateral redistribution and delay edge drainage. Flow‐probability analysis and IR thermography confirm that Stage I evaporation is edge‐dominated. With the same water input, evaporation proceeds more slowly overall for hexagonal DE‐cement tiles but keeps the surface colder for longer: after an initial period, temperatures beneath the tiles are 10–20°C lower than those beneath the cracked and non‐cracked commercial stucco tiles. Future work will integrate the measured evaporation behavior of crack‐programmed tiles with building energy modeling to quantify potential indoor cooling benefits across different climates. In addition, spray‐coating the DE‐cement mixture through a hopper gun onto mesh substrates (20″ × 25″) reproducibly generates porous‐cracked panels (Figure S23), indicating potential for large‐area, on‐site fabrication.

Together, these results deliver the combination required at building scale: fast droplet capture and rebound suppression via DE micro/nanoporosity secured by a short pre‐hydration window; programmed lateral routing that prolongs Stage I evaporation via ridge‐guided, deterministic crack lattices with an intermediate‐density hexagonal tiling; and durable, low‐complexity, scalable fabrication using embossed molds and standard curing on cementitious substrates. This work provides a simple route to creating surface structures that capture, store, and efficiently route water for passive cooling in roofs and façades. Moving forward, we envision intelligent control of watering cycles based on external weather conditions to further conserve building energy and minimize potential biofouling from excess water accumulation in cracks.

## 4 Experimental Section

### 4.1 Material Preparation

The concrete paste started with dry mixing of Ordinary Portland cement (ASTM C‐150 Type I, Quikrete, USA) and natural diatomaceous earth (DE; DiatomaceousEarth.com) at a weight ratio of 1:1. The DE used in this study was an uncalcined, naturally occurring material primarily composed of amorphous silica (>95% SiO_2_) with less than 5% crystalline silica. It was used as received, without further purification. The DE has a specific gravity of 2.2, a pH range of 7.5–9.0, low water solubility (<1%), and a melting point above 1300°C. Water was then added to the dry mix at a water/(cement+DE) mass ratio calculated by the equation: W_water_ = 0.5 × W_cement_ + 2 × W_DE_, where W_water_ is the mass of water, W_cement_ is the mass of cement, and W_DE_ is the mass of DE. After thorough mixing, the paste was cast into thin plates, either circular (3 mm thick and 150 mm in diameter) or square (3 mm thick and 100 mm × 100 mm), depending on the experiment. Samples were covered and pre‐hydrated for 1 h at room temperature and then uncovered and dried at 60°C.

### 4.2 Pre‐Hydration Test

To examine the role of pre‐hydration, six samples with 50 wt.% DE were covered and pre‐hydrated at room temperature for different durations (0, 1, 3, 6, 12, and 24 h) before drying. Crack fraction was determined using image analysis software (Image J).

### 4.3 Composition Variation Test

Eleven samples were prepared with DE mass fractions ranging from 0 to 100 wt.% in 10% increments, cast as thin circular discs (3 mm thick and 150 mm in diameter). Samples were pre‐hydrated at room temperature for 1 h and then dried in a 60°C oven to induce cracking. Top‐view images of the dried samples were used to quantify the crack area fraction as a function of DE mass fraction using image analysis software (ImageJ).

### 4.4 Physical Properties

The dry density of samples with different DE mass fractions was measured after vacuum drying at 80°C for 24 h until reaching a constant weight. The dry density was calculated as the ratio of the dry mass to the geometric volume of each sample. The specific surface area of powders obtained from dried samples was measured by N_2_ adsorption using the Brunauer‐Emmett‐Teller (BET) method. Prior to the measurement, the powders were degassed under vacuum at 120°C for 24 h to remove adsorbed moisture and gases. Nitrogen adsorption–desorption isotherms were then collected at 77 K, and the surface area was calculated from the BET analysis of the isotherm in the appropriate relative pressure range.

### 4.5 Mechanical Testing

The elastic modulus of samples with different DE mass fractions was determined by indenting the fresh mixture in a petri dish (height: 20.3 mm, diameter: 50 mm). A round‐flat end cylinder indenter with radius R = 0.5 mm was used on the Instron mechanical tester to indent the center of the sample by applying force F to a certain indentation depth δ. Elastic modulus was calculated as E = F(1‐ν^2^)/(2Rδ), where ν = 0.5 is the Poisson ratio of the fresh concrete.

### 4.6 Droplet Impact tests

Dried crack‐free samples with 0 and 50 wt.% DE were subjected to single‐droplet deposition. Water droplets were released from a height of 5 cm above the sample surface, and the impingement, spreading, recoil, and imbibition processes were recorded using a Phantom high‐speed camera (Vision Research, USA) operated at 2000 fps.

### 4.7 Water Infiltration and Flow Tests

Water addition was performed using a syringe pump connected to a fine needle to deliver a constant water flow at a fixed rate of 0.045 mL/s. For horizontal tests, water was applied at the center of the circular discs (3 mm thick and 150 mm in diameter). For tilted tests, discs were inclined at 60°, and water was applied at the top‐middle edge. In a complementary setup (Figure S12), the same syringe‐needle system was used, while an infrared camera positioned above the sample recorded water flow pathways. To enhance visualization of transport dynamics, samples were placed on a hot plate to generate a temperature contrast between the infiltrating water and the material (Figure S13). Tap water was used for all wetting, transport, and evaporative cooling experiments.

### 4.8 Substrate‐Guided Crack Programming

Molds with triangular ridges shown in Figures 4, 5, 6 were produced by 3D printing using Phrozen High‐Resolution Aqua 8K Resin. Circular samples shown in Figures 1, 2, 3, which exhibited stochastic cracking, were cast in standard Petri dishes. No release agent was used on any samples involved in the cracking tests. The number of ridges (*N* = 0–7) was systematically varied, while the total ridge volume was kept constant by adjusting the ridge tip angle (15°–45°). To evaluate water transport, water was applied at the center of the circular discs, and the propagation front was recorded sequentially. Spreading distances were measured along the ±x and ±y directions.

### 4.9 Periodic Crack Networks Design and Testing

Periodic or tessellated crack networks with triangular, square, and hexagonal unit cells were fabricated using 3D‐printed square substrate molds (3 mm thick and 100 mm × 100 mm). For each geometry, five unit‐cell sizes were designed (140.25, 364.65, 589.05, 813.45, 1037.85 mm^2^). The smallest unit cell was chosen to match the area of a single sector in the *N* = 7 radial sample (Figure 4b), which served as a benchmark for efficient stress dissipation. Larger unit cells were obtained by progressively increasing ridge spacing until random cracks began to form within the patterned domains. Water transport experiments were performed by supplying a total of 13.5 mL of water at a constant rate from a burette positioned above the sample. To avoid pooling and reabsorption, the lower substrate edge was left open and lined with a paper towel to collect the outflow (Figure S20). The change in sample mass was simultaneously recorded over time to quantify water uptake. Probability analysis was performed by applying droplets to crack intersection points, and the flow direction was recorded to determine directional spreading probabilities. The data were then combined to generate probability maps of transport across each network. Flow hierarchy was quantified by counting the number of sequential propagation levels as water advanced step by step through the cracks (Figure S21). Directional probabilities were further decomposed into horizontal and vertical directions, enabling comparison of anisotropic transport across the three geometries.

### 4.10 Image Analysis

SEM micrographs were collected using environmental scanning electron microscopy (FEI Quanta 600 ESEM). All crack‐related parameters were quantified using ImageJ (NIH, USA) from top‐view images of dried samples. Original images were first converted to grayscale and a threshold value was set to generate the binary crack masks. Crack area fraction was defined as the ratio of the crack pixels to the total projected sample area (Figure S6). The fraction of random cracks was calculated in substrate‐guided experiments by segmenting cracks not aligned with the designed paths. Crack width was measured directly from the processed images, with five positions selected along each crack to account for local variations. The reported values represent the average width calculated from these measurements.

### 4.11 Evaporative Cooling Tests

Evaporative cooling performance was evaluated using four square samples (3 mm thick and 100 mm × 100 mm): (i) a crack‐free DE‐cement control, (ii) a hexagonal crack network with the smallest unit cell size, (iii) a hexagonal crack network with an intermediate unit cell size, and (iv) a hexagonal crack network with the largest unit cell size. All samples were loaded with equal water volumes and tested under natural convection at constant ambient temperature (∼23°C) and relative humidity (25%). The initial specimen surface temperature was equilibrated to ∼21°C prior to dosing. Surface cooling and spatial temperature distributions were monitored by infrared (IR) thermography (Figure 6a; Figure S24), while the continuous mass loss was recorded using a precision balance to quantify evaporation rates (Figure 6c).

### 4.12 Model Roof Demonstration

A scaled bench‐top model home (base dimensions: 30 cm × 15 cm, house height: 15 cm) with a roof angle of 60° was assembled to evaluate the evaporative cooling performance of the DE‐cement tiles. Three types of tiles (150 mm×100 mm×3 mm) were prepared: (i) DE‐cement composites containing 50 wt.% DE with intermediate‐density hexagonal crack patterns, (ii) commercial stucco (Stucco Patch, Rapid Set) tiles with the same crack geometry, and (iii) non‐cracked stucco tiles as controls. The tiles were mounted on expanded polystyrene (EPS) roof bases that provided thermal insulation and structural support. Each EPS base contained a small central opening directly beneath the tile to accommodate a K‐type thermocouple, enabling real‐time measurement of the transmitted temperature through the sample. A second thermocouple was positioned on the top surface, near the center, to monitor the incident temperature from the heat source, which was maintained at 42 ± 1°C. Temperature data were collected using a digital data logger at 10 s intervals throughout the experiment. An infrared (IR) lamp positioned at a fixed distance (200 mm) above the sample surface served as the heat source, ensuring uniform irradiation over the tile area. Water was supplied at the roof ridge every 30 min using a burette at a constant flow rate, with a total of 15.5 g per dosing cycle, to simulate periodic watering. Each test lasted 3 h (six watering cycles). All experiments were conducted under ambient laboratory conditions (temperature: 24 ± 1°C, relative humidity: 40%–45%). The recorded temperature beneath each sample was used to evaluate the cooling performance by comparing the steady‐state and transient temperature profiles among the DE‐cement, cracked stucco, and non‐cracked stucco tiles.

## Author Contributions

Q.H., K.Y., and S.Y. conceived the idea. Q.H., K.Y., J.B., Y.L., D.A., and S.Y. designed the research and interpreted the results. Q.H., K.Y., and J.B. conducted the experiments. Q.H., K.Y., J.B., D.A., and S.Y. wrote the manuscript. All authors contributed to revising the manuscript.

## Conflicts of Interest

The authors declare no conflict of interest.

## Supporting information




**Supporting File 1**: adma73109‐sup‐0001‐SuppMat.pdf.

**Supporting File 2**: adma73109‐sup‐0002‐MovieS1‐S8.zip.

## Data Availability

The data that support the findings of this study are available in the supplementary material of this article.
