# Supplementary material for: Elephant‐Skin‐Inspired Porous Cementitious Tiles with Programmable Crack Networks for Passive Cooling
Source: Adv Mater. 2026 Apr 20;38(44):e23133. doi: 10.1002/adma.202523133 (PMC13449127; doi:10.1002/adma.202523133)
Supplement: Supplementary file 1 [file ADMA-38-e23133-s002.pdf]

Supplementary Information

Elephant-Skin-Inspired Porous Cementitious Tiles with Programmable Crack Networks for Passive Cooling

Qingya Huang[1,3], Kun-Hao Yu[1,3,*], Ji Yoon Bae[2], Yunchan Lee[1], Dorit Aviv[2], Shu Yang[1,*]

[1] Department of Materials Science and Engineering, University of Pennsylvania, 3231 Walnut Street, Philadelphia, PA 19104, USA

[2] Department of Architecture, Weitzman School of Design, University of Pennsylvania, 210 S 34th St, Philadelphia, PA 19104, USA

[3] These authors contributed equally: Qingya Huang and Kun-Hao Yu

[*] Correspondence to shuyang@seas.upenn.edu and kyu131@syr.edu

This PDF file includes:

Figure S1 to S24

Video S1 to S8 Captions

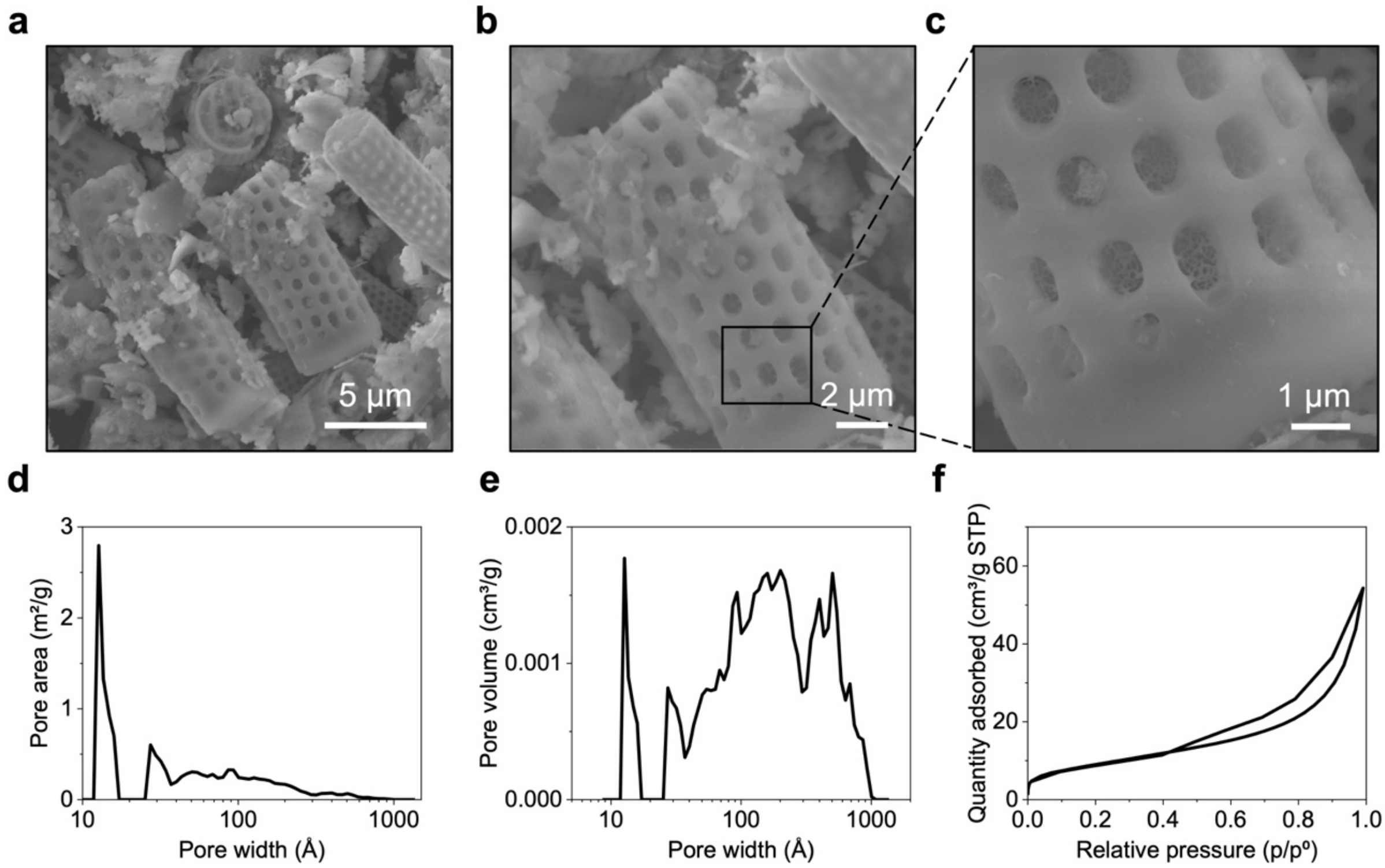

Figure S1. Characterization of diatomaceous earth (DE). a–c) SEM images of DE at increasing magnification. d) Pore-area distribution. e) Pore-volume distribution. f) N_2 adsorption-desorption isotherm.

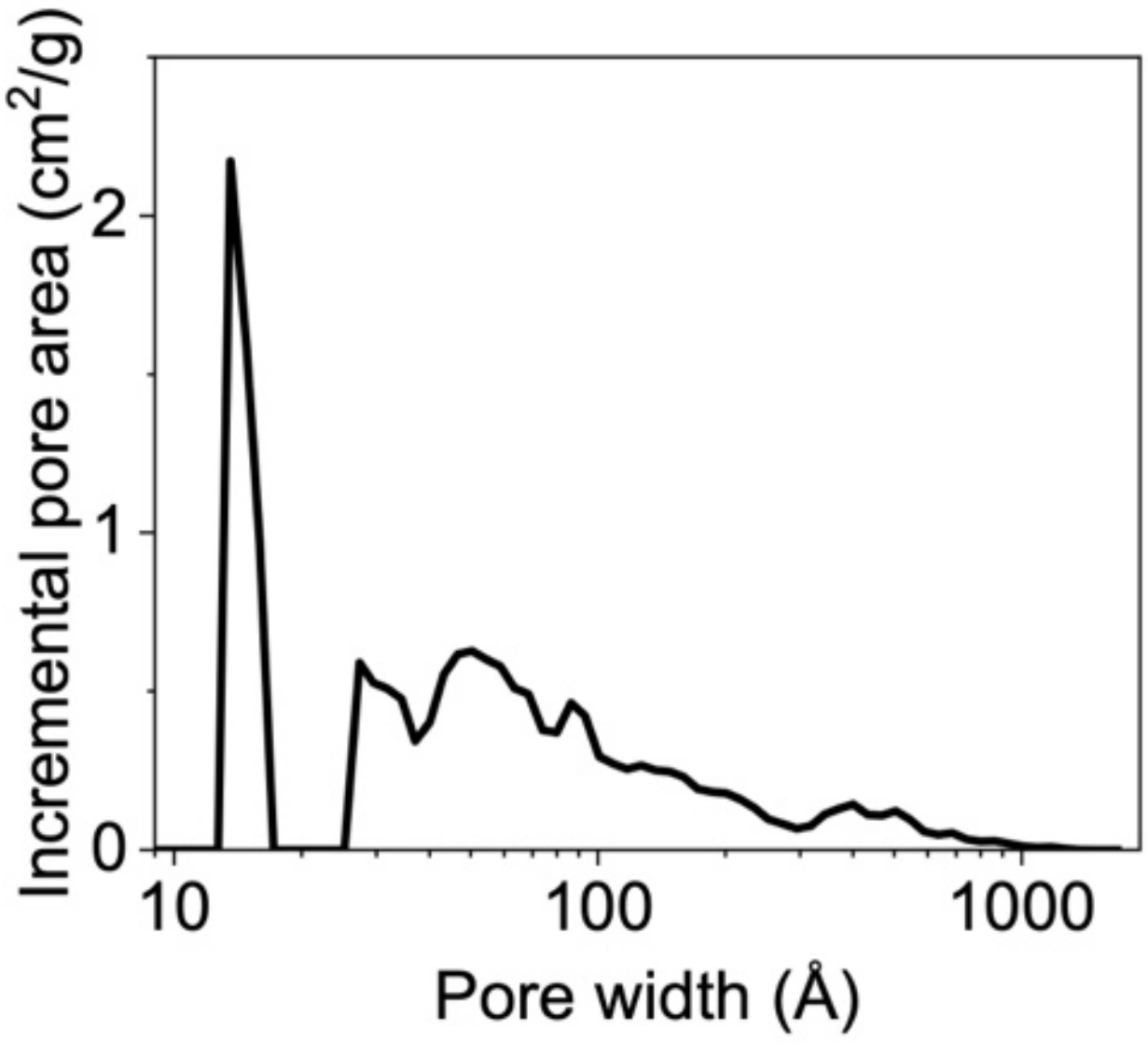

Figure S2. Pore area distribution of the DE-cement composite.

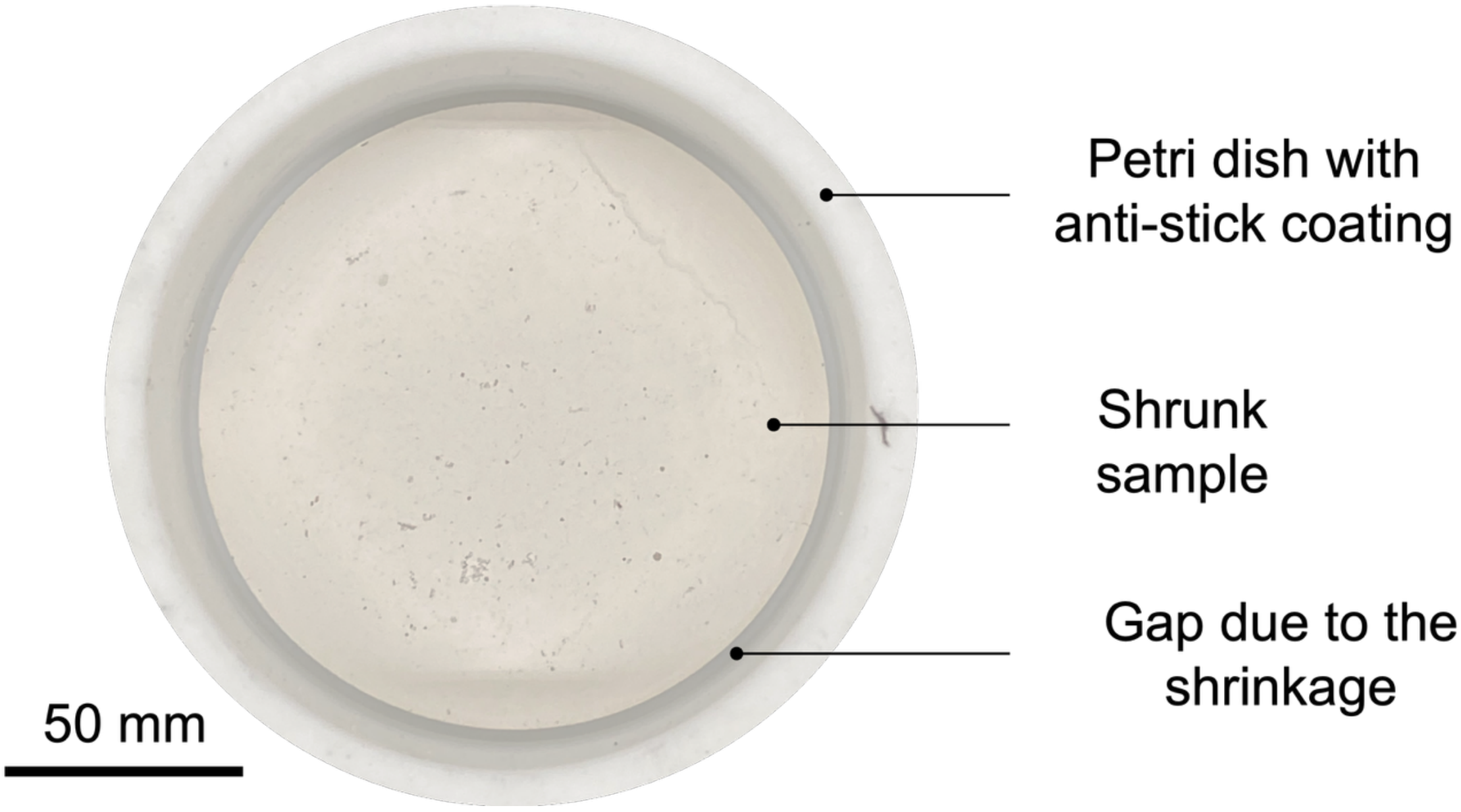

Figure S3. Image of a 50 wt% DE sample dried on a substrate coated with a non-stick lubricant, showing free shrinkage without cracks.

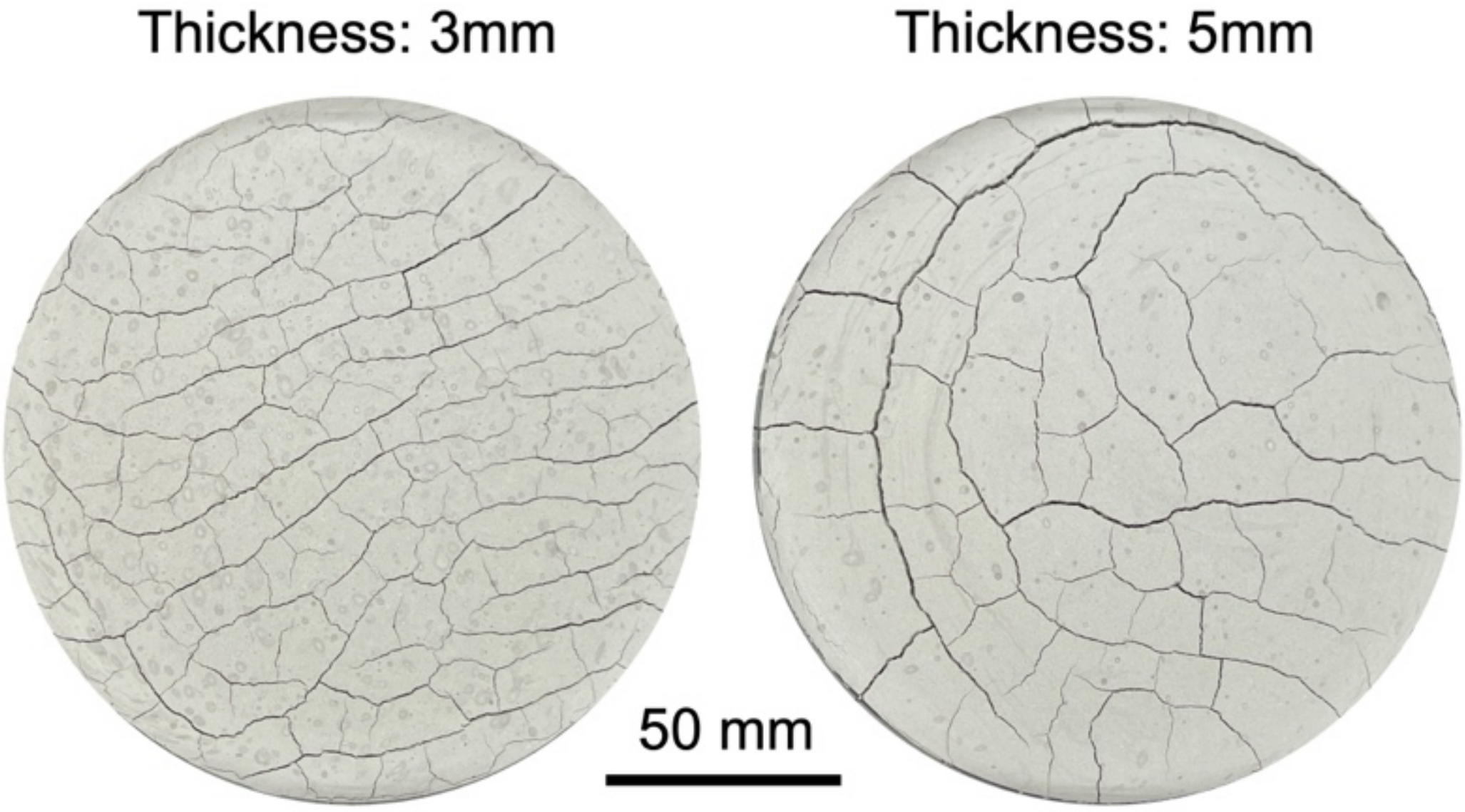

Figure S4. Effect of sample thickness on fragment size and crack area fraction for 50 wt% DE composite discs.

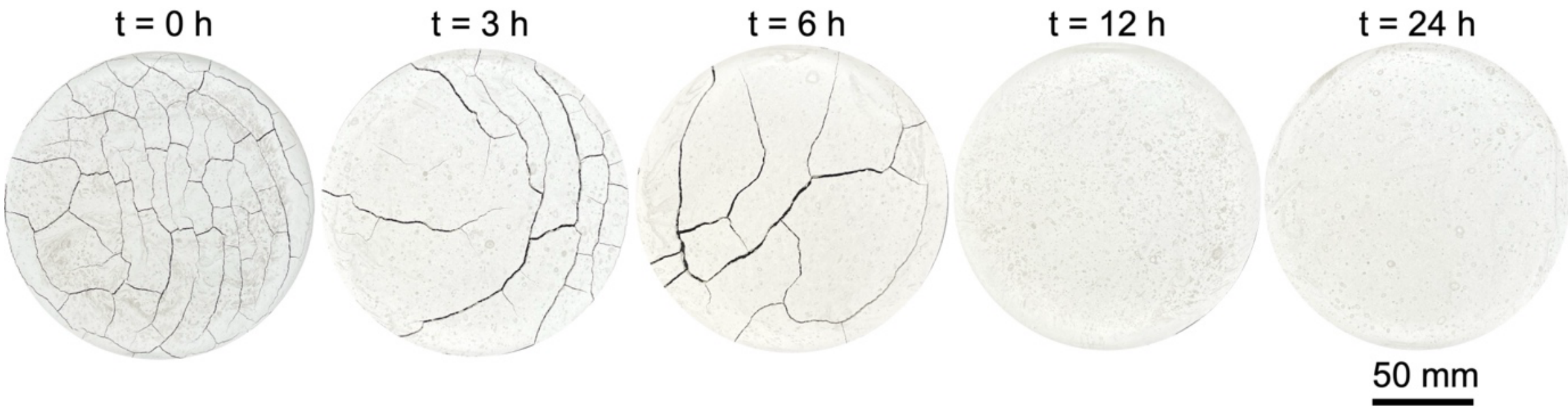

Figure S5. Effect of pre-hydration duration on crack formation for 50 wt% DE composite discs.

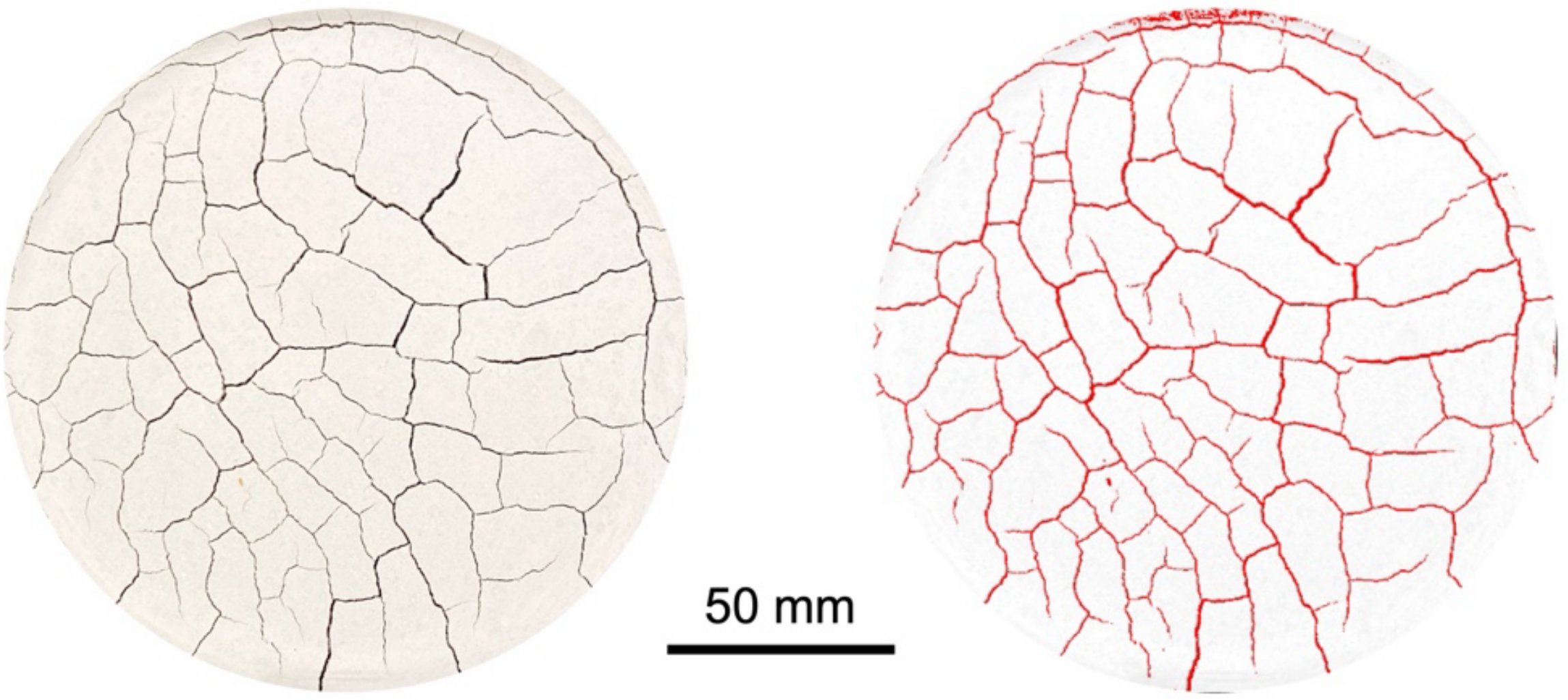

Figure S6. Image analysis (Right) of the DE-cement sample (Left) for quantifying crack area fraction.

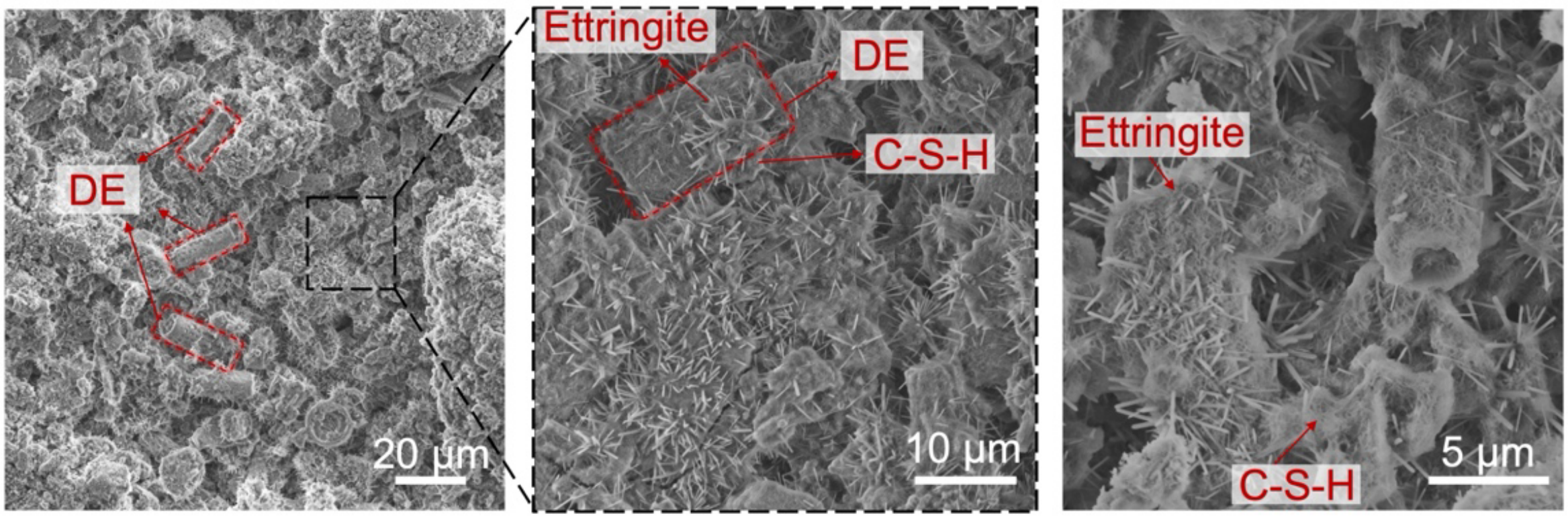

Figure S7. SEM images of the 50 wt% DE composite after 24 h pre-hydration showing C-S-H/ettringite bridging at DE interfaces (DE outline in red).

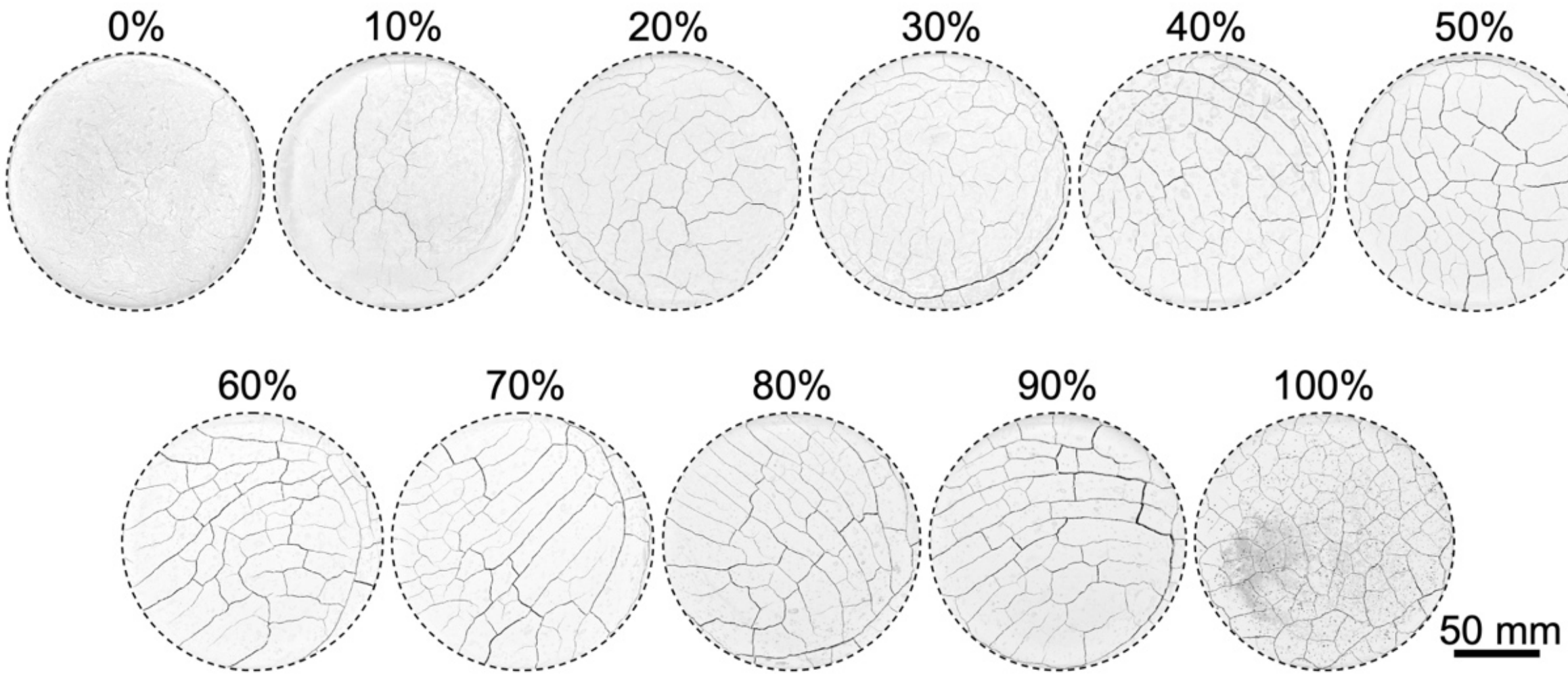

Figure S8. Effect of DE mass fraction in the composite on crack formation.

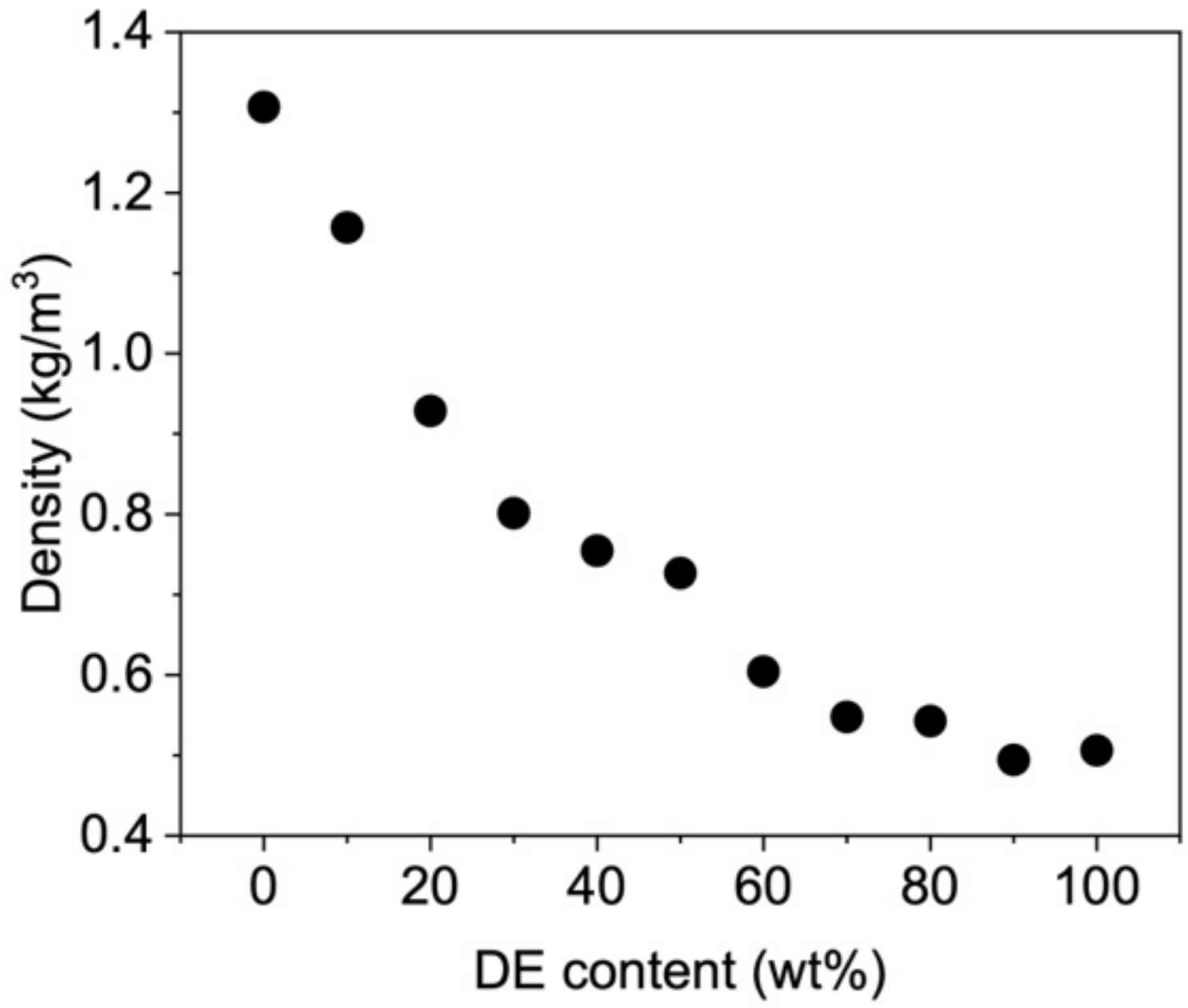

Figure S9. Dry bulk density versus DE mass fraction in the composites.

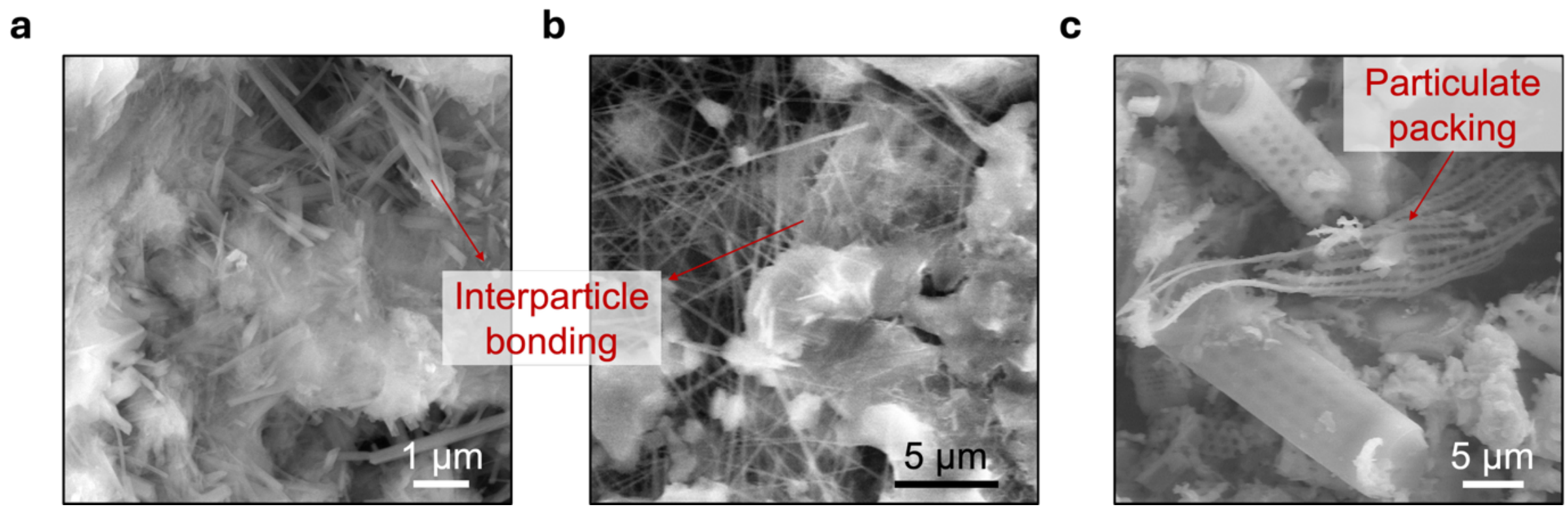

Figure S10. SEM images of the DE-cement composites of different DE mass fraction. a) 0 wt% DE, b) 50 wt% DE, and c) 100 wt% DE, highlighted with interparticle bonding versus particulate packing.

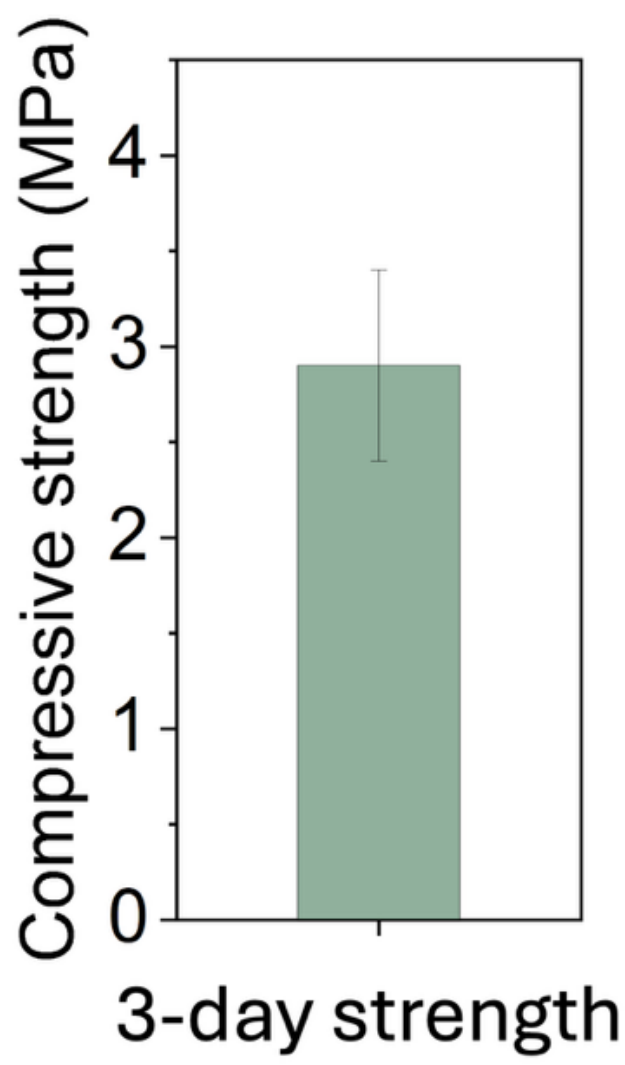

Figure S11. Compressive strength of the 50% DE-cement composite after curing for 3 days.

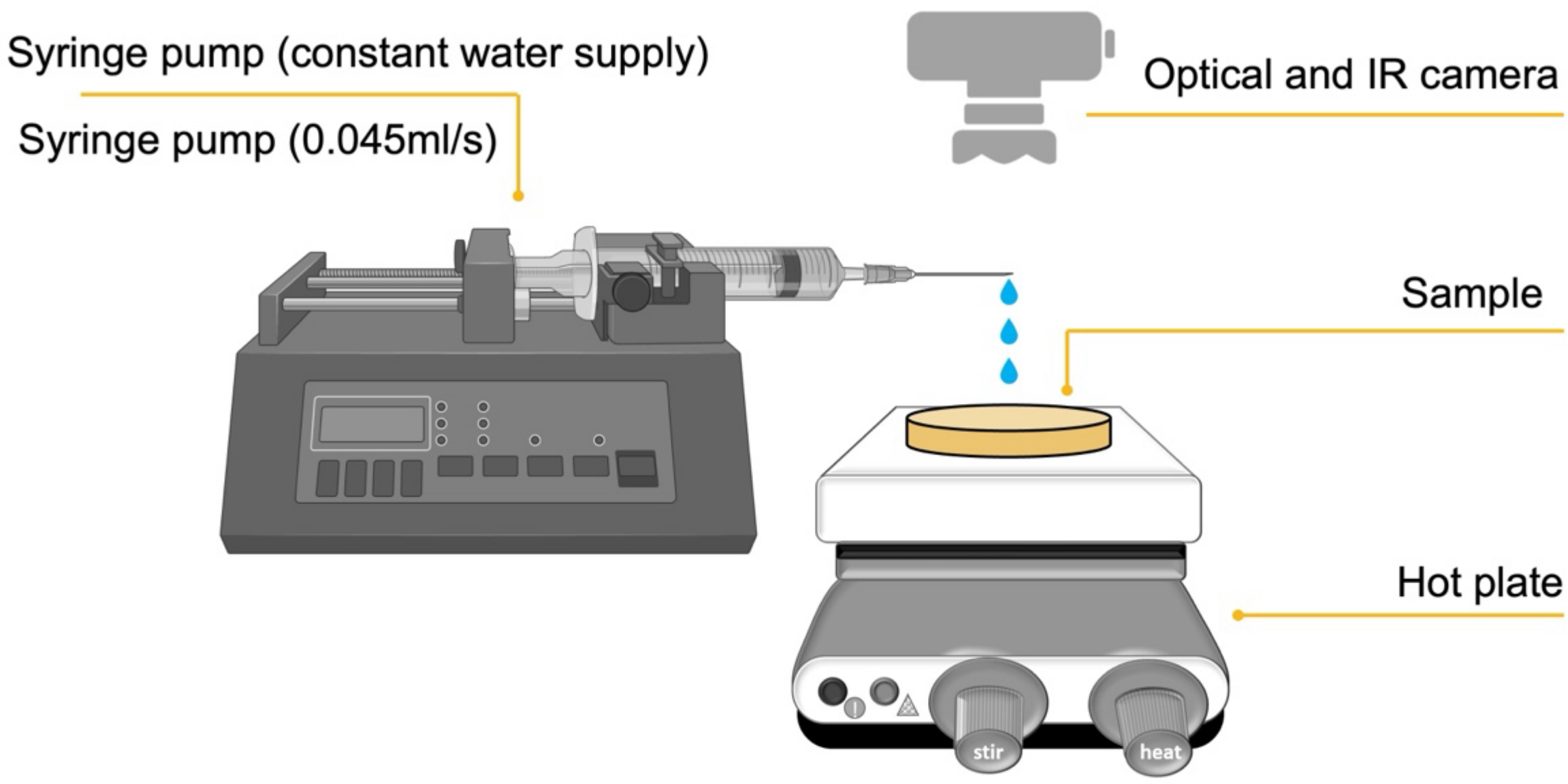

Figure S12. Schematic of the setup for continuous-flow/IR visualization.

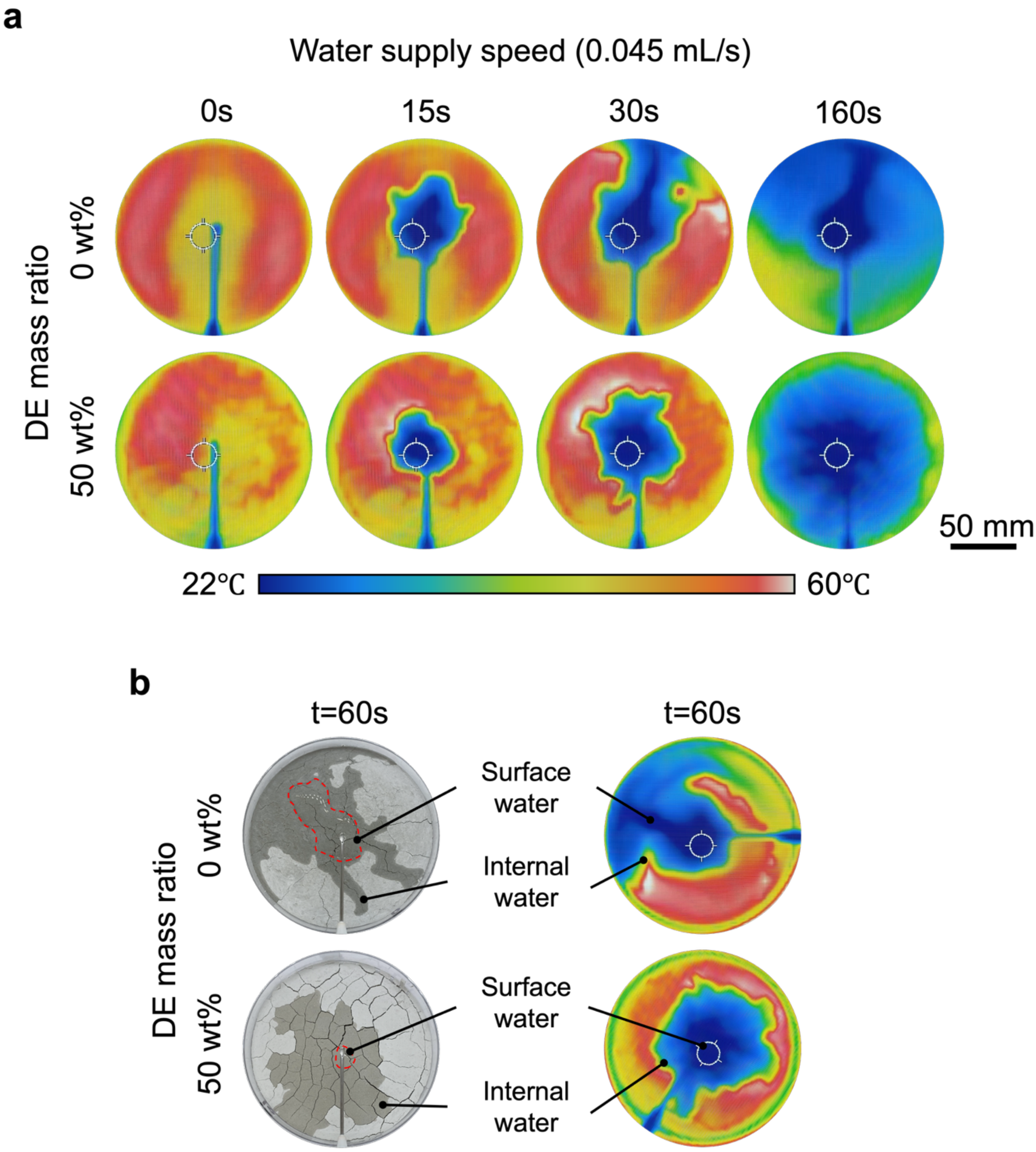

Figure S13. Images of water transported at various time points for samples with 0 and 50 wt% DE, respectively. (a) IR images of water transported at 0s, 15s, 30s, and 160s. (b) Optical and IR images of water transported at 60s, showing the locations of surface and internal water.

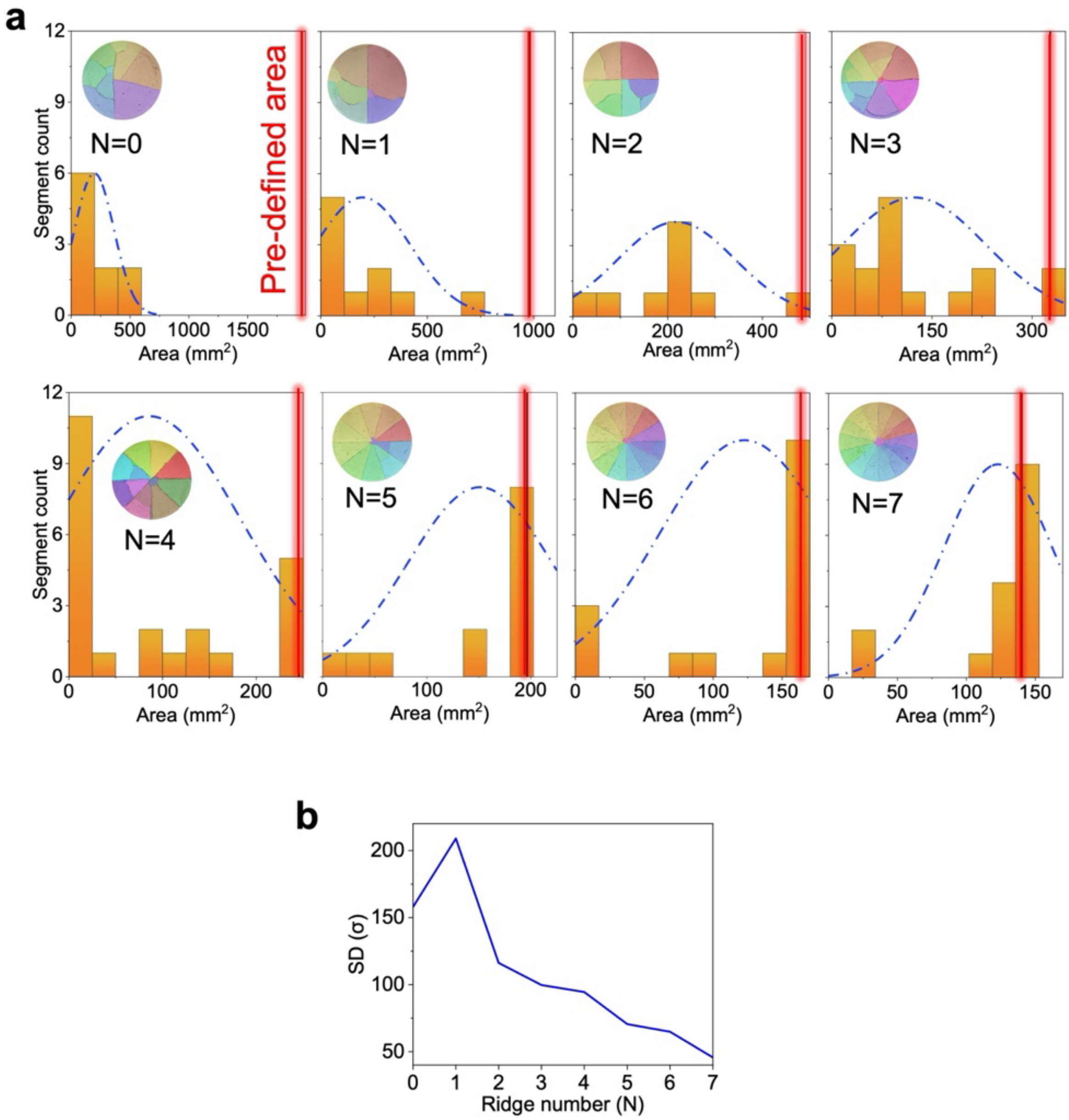

Figure S14. Uniformity of the ridge-guided segmentation. a) Distributions of the crack-segment areas for N = 0–7 (red line: pre-defined segment area from ridge geometry). b) Standard deviation of the segment areas versus *N*.

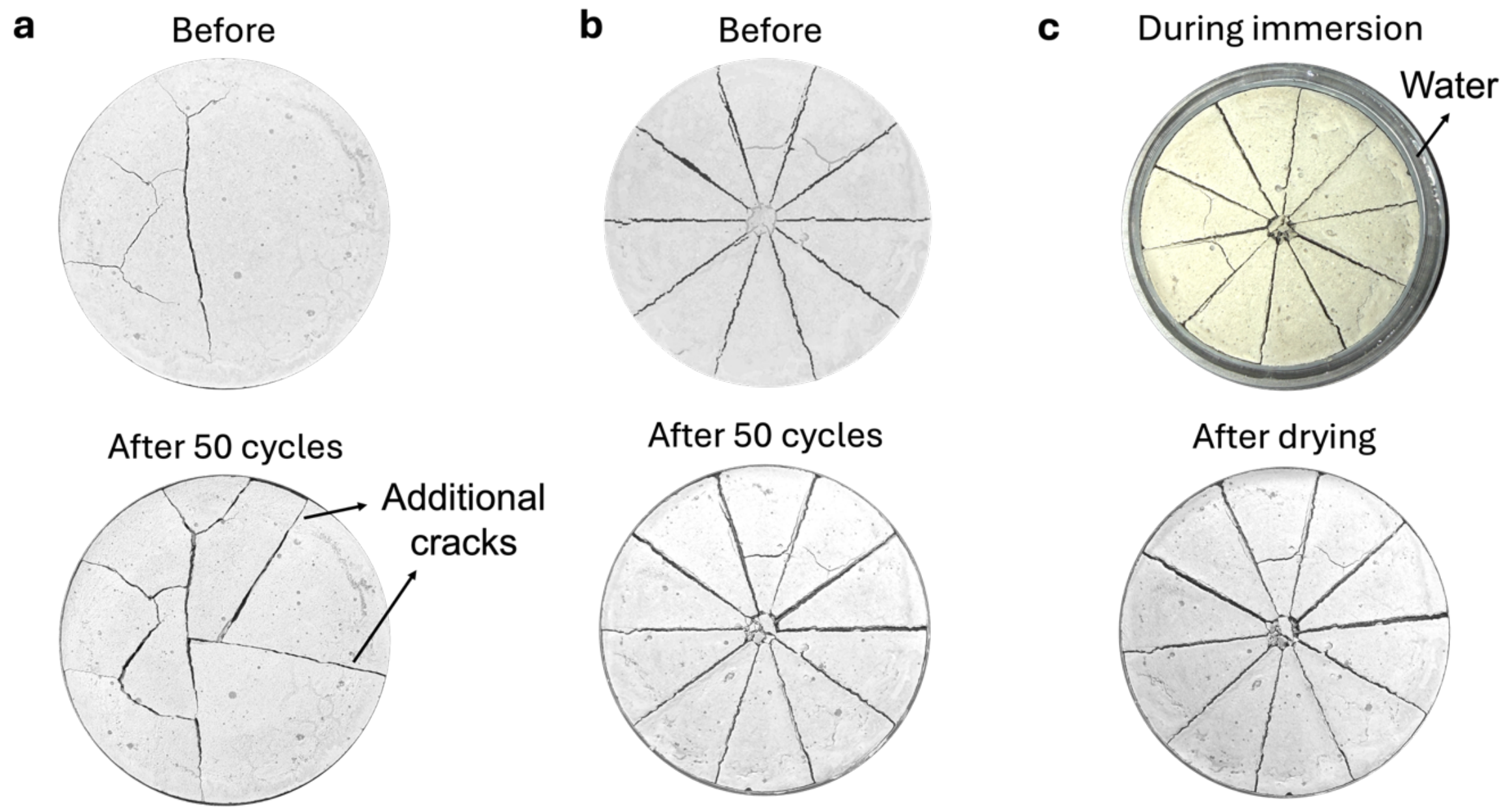

Figure S15. Photographs of the crack networks under repeated wetting-drying cycles and immersion-drying tests. (a) A non-patterned sample (N = 0) before and after 50 wet-dry cycles. (b) A ridge-patterned sample (N = 5) before and after 50 wet–dry cycles. (c) A ridge-patterned sample during 24 h immersion in water, followed by drying in air.

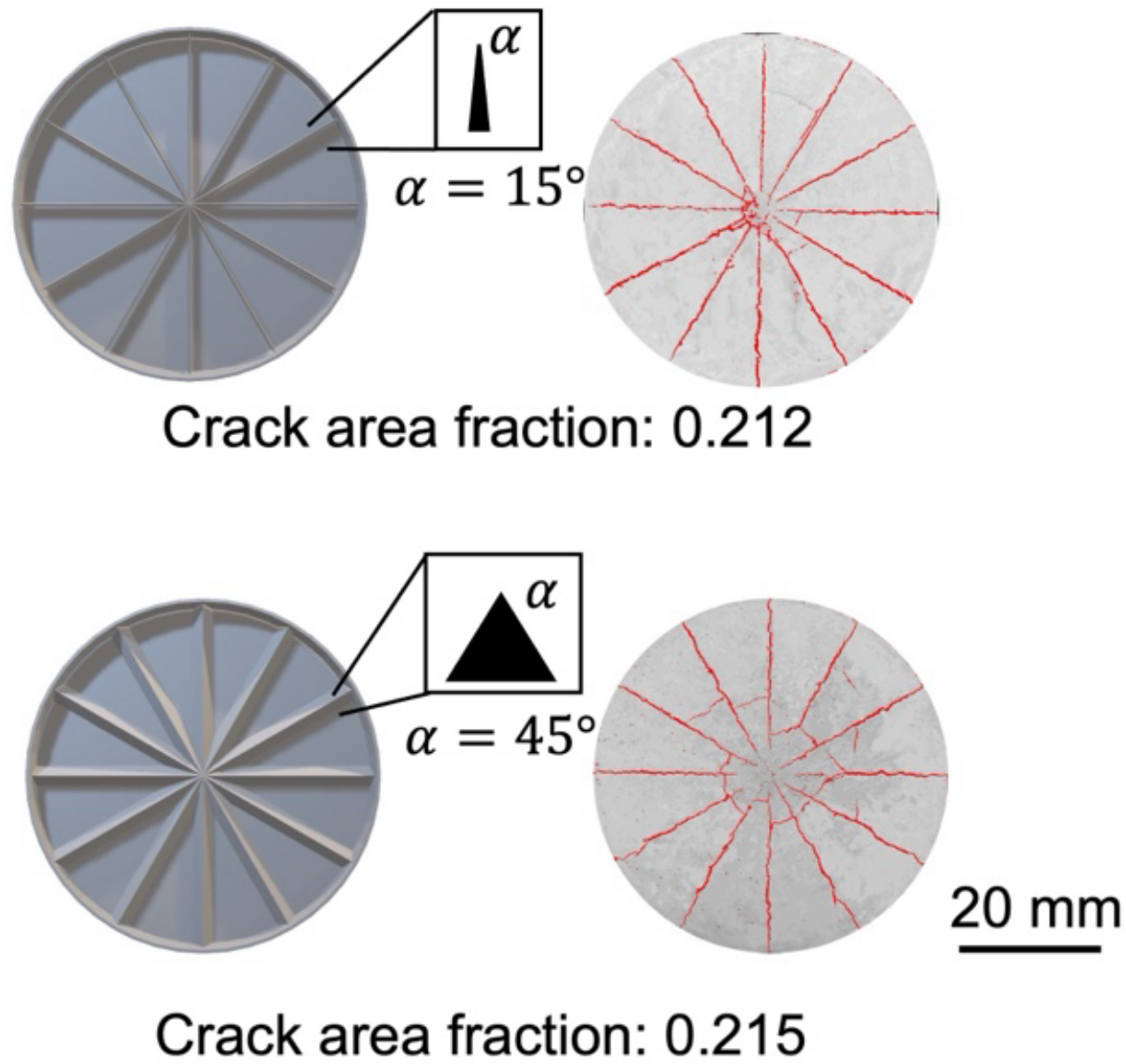

Figure S16. Effect of ridge tip angle. Crack patterns generated in the composite discs (50 wt% DE) from substrates (*N* = 6) with the ridge tip angles (α) of 15° and 45°, respectively, showing robust guidance by the substrate.

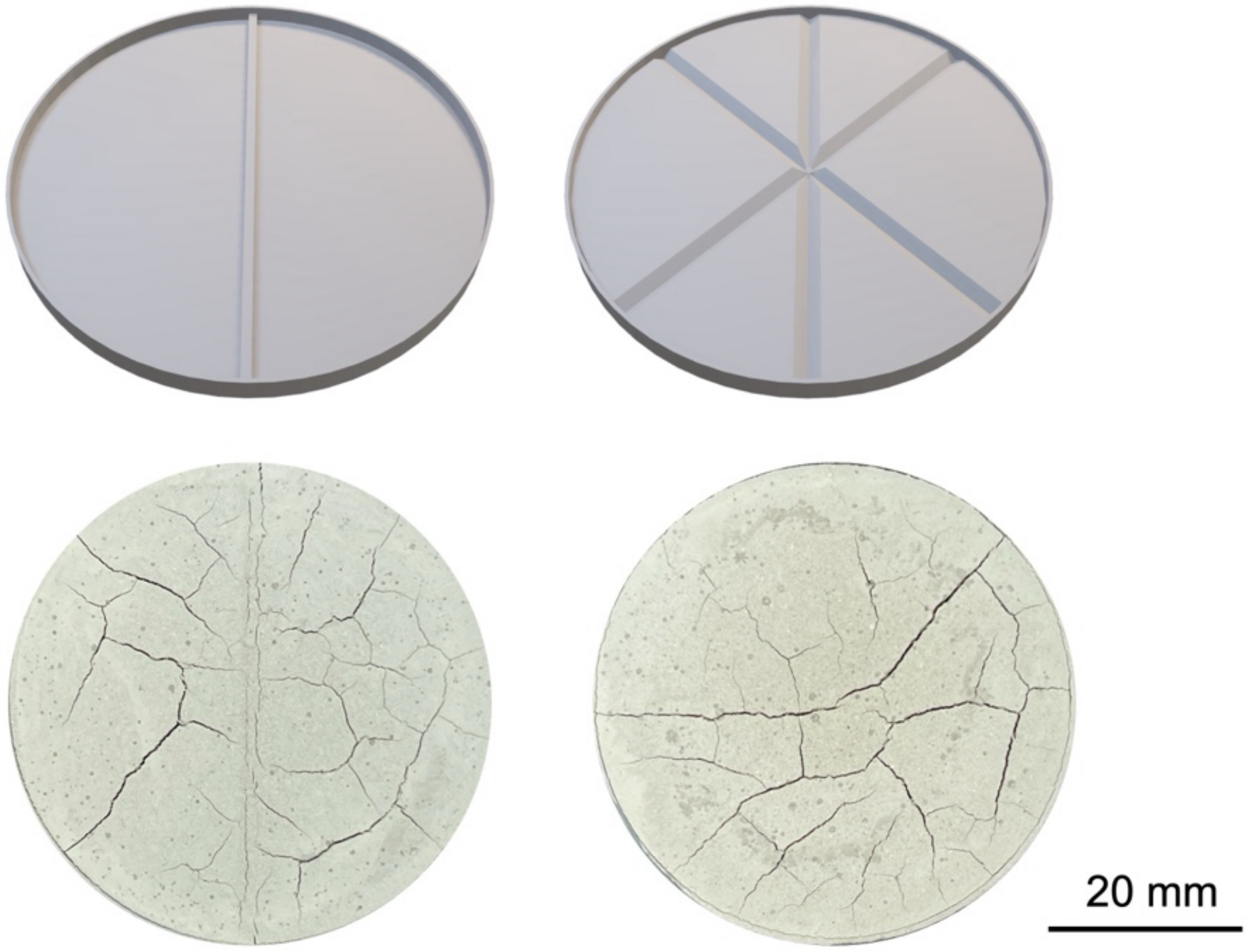

Figure S17. Crack formation on substrates with rectangular features and concave triangular (V-shaped) grooves, showing no reliable localization of crack initiation.

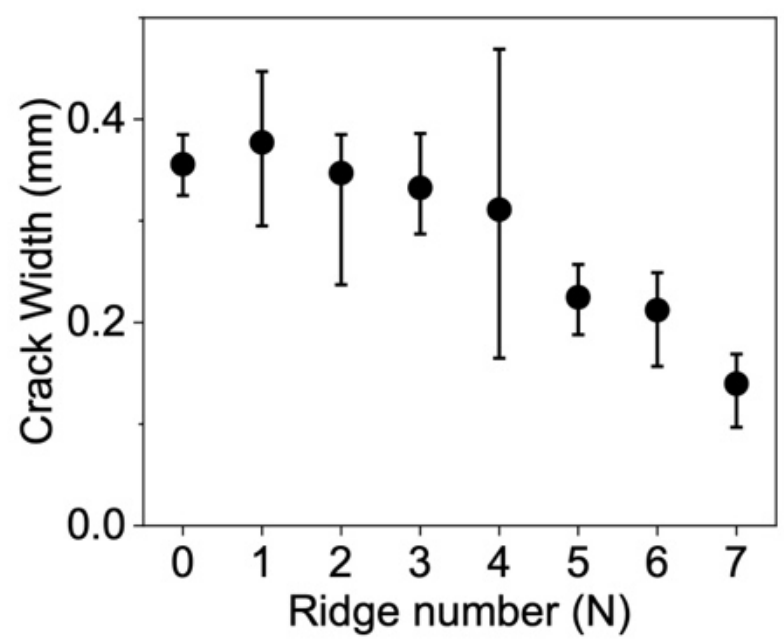

Figure S18. Effect of ridge number versus crack width for 50 wt% DE sample. Each crack's width is averaged over 5 different points.

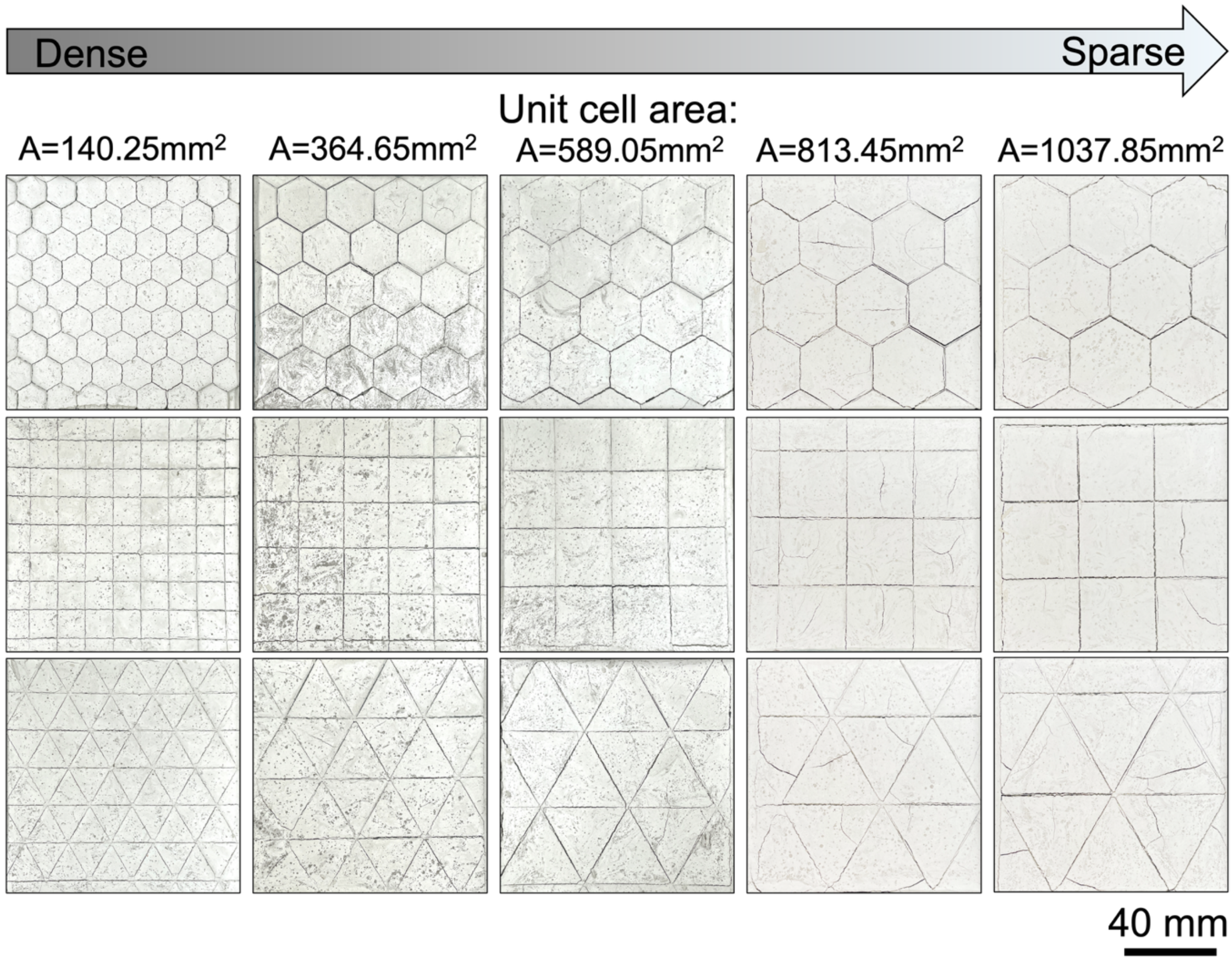

Figure S19. Square tiles patterned with triangular, square, and hexagonal networks across five unit-cell areas per geometry.

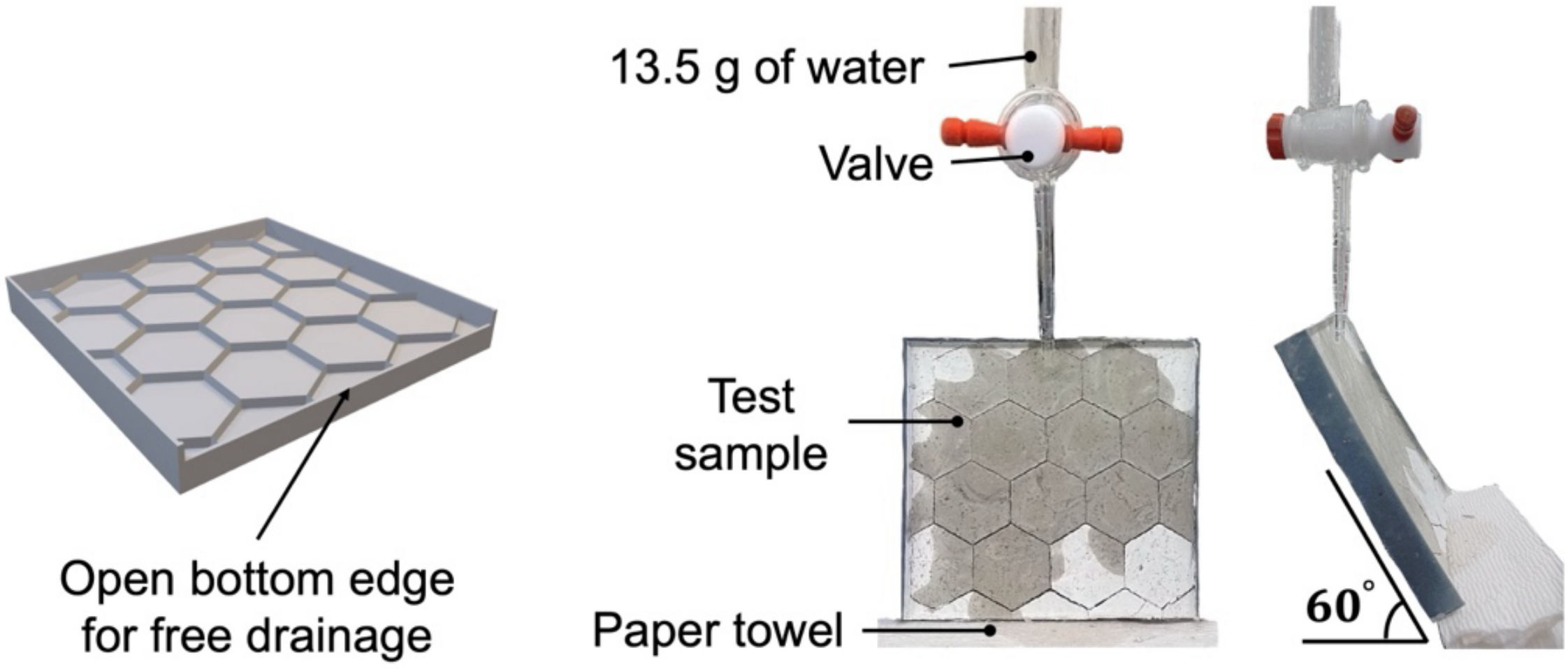

Figure S20. Experimental setup and substrate design for tilted water-uptake tests. The bottom edge of the substrate is removed to avoid reabsorption from pooling.

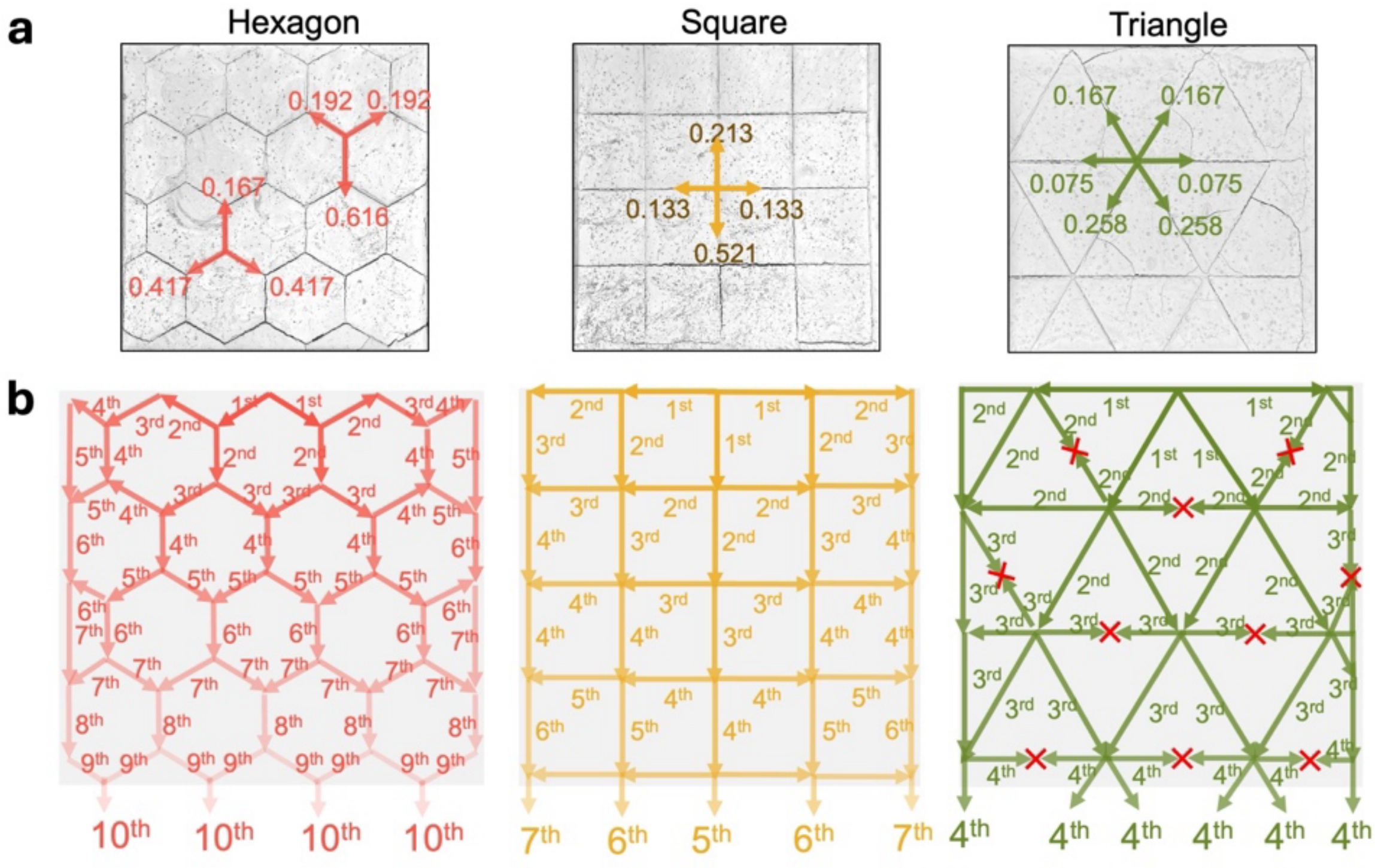

Figure S21. Directional transport analysis and simulations. (a) Measured directional flow probabilities at intersections for hexagonal, square, and triangular networks at a 60° tilt angle. The numbers adjacent to each arrow represent the probability of water moving in that direction at a junction, obtained by averaging 10 experimental observations across multiple samples. (b) Simulated stepwise movement (random walks) starting at the top center; each step corresponds to motion from one intersection to the next until exiting at the bottom edge.

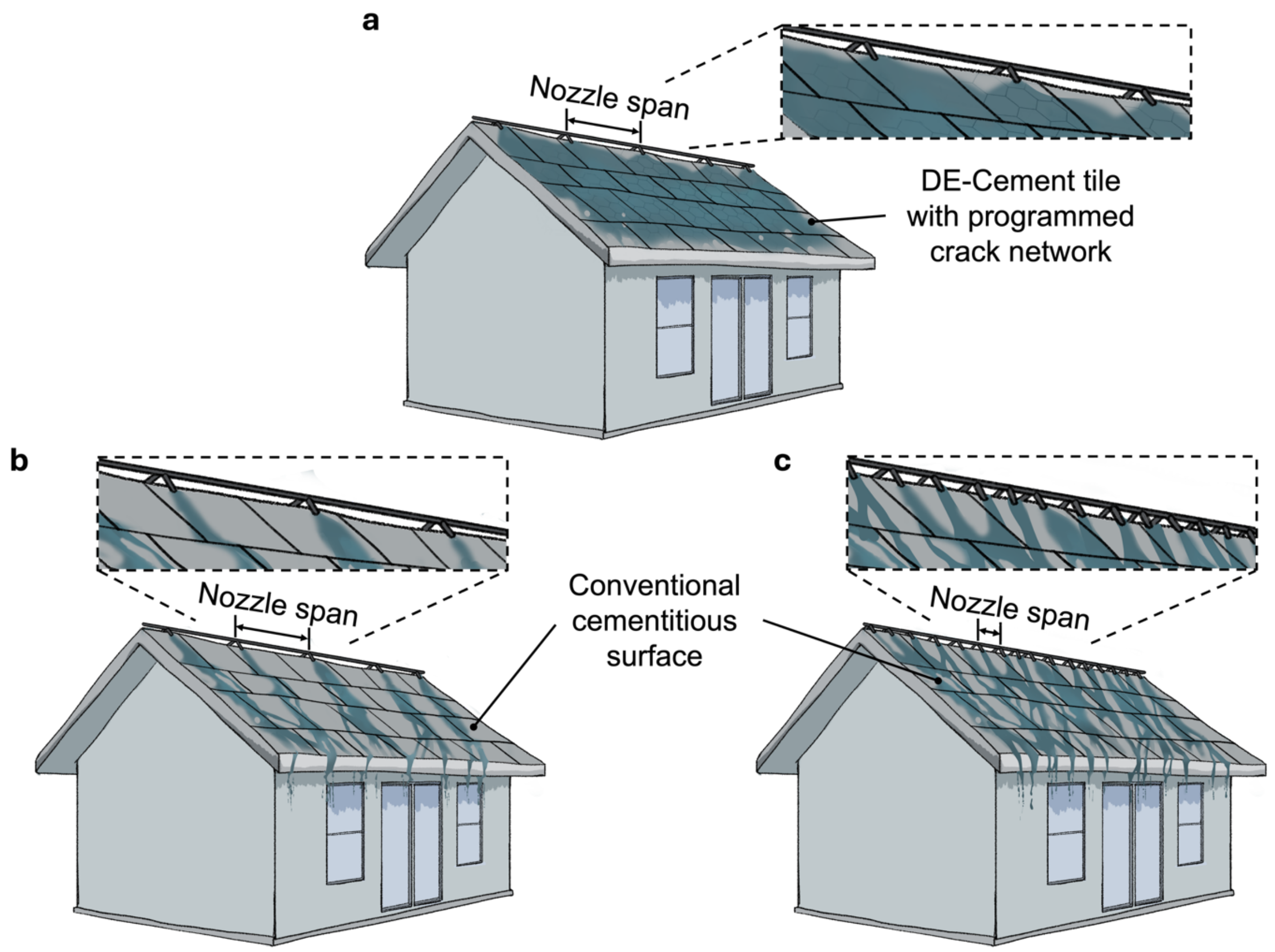

Figure S22. Conceptual illustration of representative roof-scale water-feeding configurations. a) DE-cement tiles with programmed crack networks under ridge-mounted water supply. b) Conventional cementitious surface under the same nozzle spacing. c) Conventional surface requiring closer nozzle spacing to achieve comparable surface wetting.

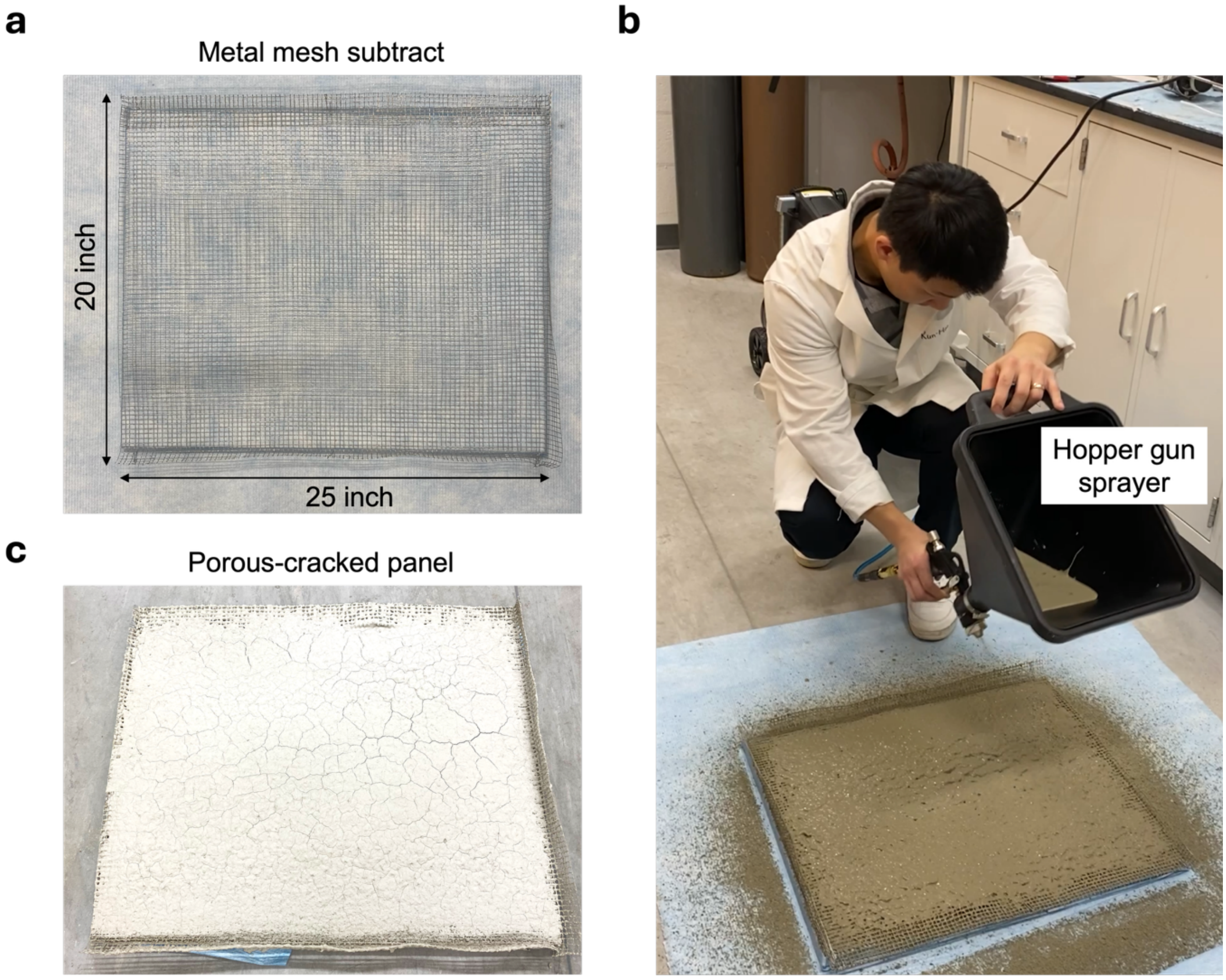

Figure S23. Large-area spray coating demonstration. a) Metal mesh substrate (20″ × 25″) used for spray coating. b) Spray-coating process of the DE-cement mixture using a hopper gun sprayer. c) Resulting porous-cracked coating formed after drying.

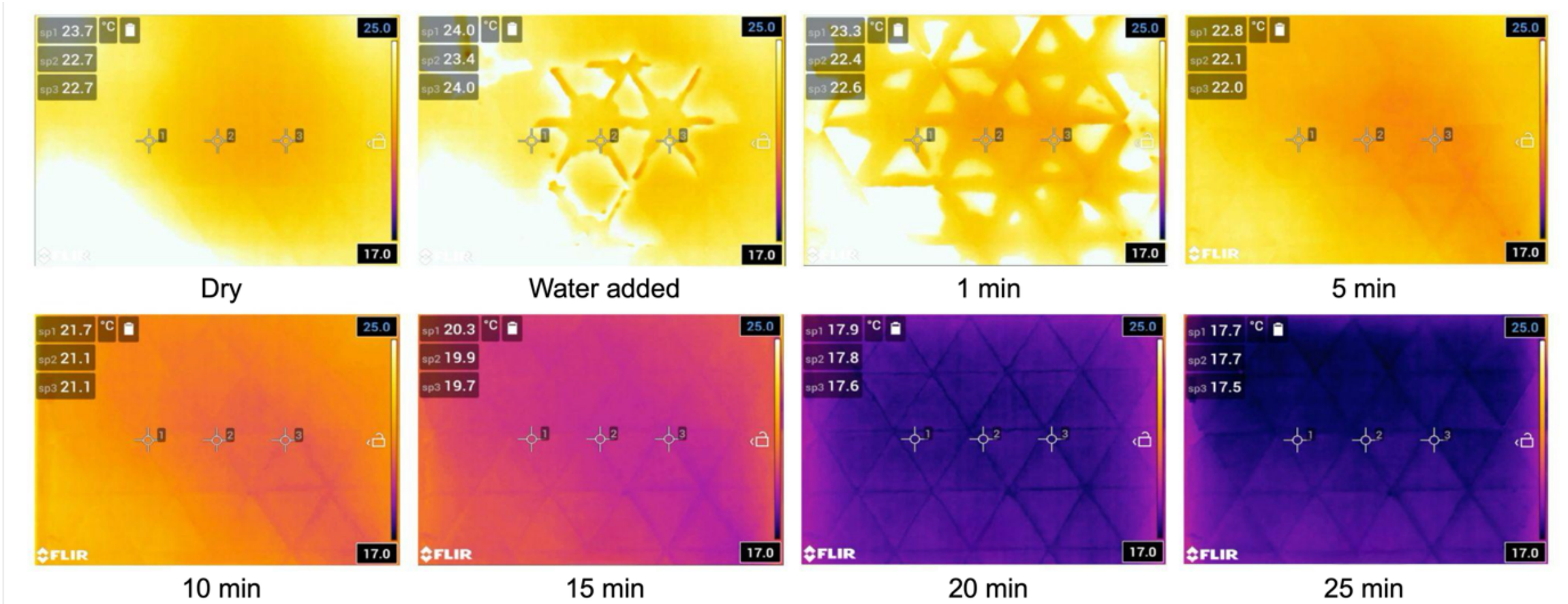

Figure S24. Time-resolved IR thermography images of the tile surface during the evaporative cooling experiment. Images correspond to the dry state, immediately after water dosing, and during drying at 1, 5, 10, 15, 20, and 25 min after dosing, respectively.

Supplementary videos captions

Video S1: Spray-on wetting of a cracked DE-cement disc.

Video S2: High-speed recording of single-droplet impact on non-cracked regions of 0 wt% and 50 wt% DE-cement discs.

Video S3: Continuous central water dosing on cracked discs composed of 0 wt% and 50 wt% DE-cement.

Video S4: Infrared imaging during continuous central room-temperature water dosing on 60 °C cracked discs (0 wt% and 50 wt% DE-cement).

Video S5: Continuous water dosing on cracked discs (0 wt% and 50 wt% DE–cement) at a 60° incline.

Video S6: Continuous central dosing on unprogrammed (N=0) and radial-programmed (N=5) crack networks.

Video S7: Continuous central dosing on programmed crack geometries: parallel, hybrid (radial & parallel), and concentric patterns.

Video S8: Equal-volume dosing on 60°-inclined periodic lattices (triangular, square, hexagonal) across multiple unit-cell sizes.
